# Modeling oxaliplatin resistance in colorectal cancer reveals a *SERPINE1*-based gene signature (RESIST-M) and therapeutic strategies for pro-metastatic CMS4 subtype

**DOI:** 10.1038/s41419-025-07855-y

**Published:** 2025-07-16

**Authors:** Stephen Qi Rong Wong, Mohua Das, Kenzom Tenzin, Niranjan Shirgaonkar, Huiwen Chua, Lin Xuan Chee, Ahpa Sae Yeoh, Astley Aruna Murugiah, Wei Yong Chua, Madelaine Skolastika Theardy, Ethan Jadon Subel, Matan Thangavelu Thangavelu, Jane Vin Chan, Choon Kong Yap, Iain Bee Huat Tan, Petros Tsantoulis, Sabine Tejpar, Jia Min Loo, Ramanuj DasGupta

**Affiliations:** 1 Genome Institute of Singapore, Laboratory of Precision Oncology and Cancer Evolution, Agency for Science, Technology and Research, Singapore, Singapore; 2 Department of Urology, Methodist Neal Cancer Center, Houston Methodist Research Institute, Houston, TX USA; 3 EDDC Academic Research Organization, Agency for Science, Technology and Research, Singapore, Singapore; 4 National Cancer Center Singapore, Laboratory of Translational Research in Gastrointestinal Cancer, Singapore, Singapore; 5 Centre for Translational Research in Onco-Hematology, Faculty of Medicine, University of Geneva, and Swiss Cancer Centre Leman, Geneva, Switzerland; 6 Molecular Digestive Oncology, Department of Oncology, Katholieke Universiteit Leuven, Leuven, Belgium; 7 CRUK Scotland Institute, School of Cancer Sciences, University of Glasgow, Garscube Estate, Glasgow, Scotland; 8 Present Address: Department of Urology, Methodist Neal Cancer Center, Houston Methodist Research Institute, Houston, TX USA

**Keywords:** Colorectal cancer, Metastasis, High-throughput screening, Mechanisms of disease, Cancer genomics

## Abstract

Drug resistance and distant metastases are leading causes of mortality in colorectal cancer (CRC), yet the molecular mechanisms linking these processes remain elusive. In this study, we demonstrate that acquired resistance to oxaliplatin, a first-line chemotherapeutic in CRC, enhances metastatic potential through transcriptional reprogramming. Using a clinically relevant dosing regimen, we generated oxaliplatin-resistant CRC cells that displayed increased metastatic potential. Integrated transcriptomic and phenotypic analyses revealed that dysregulated cholesterol biogenesis amplifies TGF-β signaling, which in turn drives expression of *SERPINE1*, which serves as a key effector of both oxaliplatin resistance and metastasis. Furthermore, we uncovered a *SERPINE1*-associated nine-gene expression signature, RESIST-M, that robustly predicts overall and relapse-free survival across distinct patient cohorts. Notably, RESIST-M stratifies a high-risk subtype of CMS4/iCMS3-fibrotic patients that display the poorest prognosis, underscoring its clinical relevance. Targeting of *SERPINE1* or cholesterol biosynthesis re-sensitized resistant, pro-metastatic cells to oxaliplatin in mouse xenograft models. Altogether, this study uncovers a mechanistic link between metabolic rewiring and transcriptional plasticity underlying therapy-induced metastasis in primary CRC. Additionally, it also reveals actionable vulnerabilities that offer both prognostic value and therapeutic potential.

## Introduction

Colorectal cancer (CRC) remains one of the leading causes of cancer-related mortality worldwide. While over 25% of CRC patients are diagnosed with distant metastatic disease at presentation, an estimated 40–50% of patients will eventually develop metastases over the course of neoadjuvant or post-surgical adjuvant therapy [1, 2]. This suggests the presence of previously disseminated cancer cells at distal sites, primarily in the liver or lungs, that are either inherently resistant to chemotherapy or acquire resistance during treatment. The 5-year survival rate for patients with unresectable metastatic CRC remains a dismal 15% [1, 2], highlighting the need to identify molecular mechanisms driving therapeutic resistance and metastatic relapse.

Despite advancements in targeted therapies and immunotherapy, combinatorial adjuvant chemotherapy, such as FOLFOX and FOLFIRI, remains the cornerstone of CRC treatment. The MOSAIC [3] and NSABP C-07 [4] clinical trials established oxaliplatin as a standard adjuvant therapy for resected stage II/III colon cancer, showing that its addition to 5-fluorouracil (5-FU) and leucovorin (LV) significantly improves disease-free survival (DFS) [3, 4]. However, long-term follow-up from the MOSAIC trial revealed that the absolute overall survival (OS) benefit for stage III CRC patients receiving oxaliplatin was a modest 4.6% over 10 years. Notably, patients with the stem-like consensus molecular subtype 4 (CMS-4), which is linked to poor prognoses, benefited the least from oxaliplatin in the NSABP C-07 and MOSAIC trials [5]. These findings underscore the need to better stratify stage II/III patients, not only to identify those at high risk to relapse, but more importantly, who is most likely to benefit from oxaliplatin therapy.

Mechanisms of oxaliplatin resistance in cancer cells have been extensively studied [6]. Recent research has highlighted several key contributors to acquired resistance, including cell cycle-dependent kinase (CDK1) [7], pyruvate kinase M2 (PKM2) [8], and various drug transporters [9]. Additionally, the activation of CD95 has been implicated in enhancing the metastatic potential of oxaliplatin-resistant cells by promoting epithelial-to-mesenchymal transition (EMT) [10]. Notably, inhibiting glutathione synthesis has been shown to sensitize peritoneal metastases in CRC patient-derived organoids (PDOs) to oxaliplatin treatment [11], emphasizing the importance of targeting acquired vulnerabilities in resistant metastatic disease. Despite these advancements, a systematic and clinically relevant understanding of how oxaliplatin-resistant CRC cells acquire metastatic traits and how these adaptations impact patient prognosis remains relatively underexplored.

In this study, we modeled CRC progression to metastatic disease following treatment of two different CRC cell lines, HCT116 and SW480, with clinically relevant doses of oxaliplatin. Molecular and phenotypic characterization revealed the activation of a cholesterol-TGF-β-*SERPINE1* signaling axis during treatment, which appeared to drive both oxaliplatin resistance and enhanced metastatic potential, specifically in the HCT116 cell line. Through transcriptomic profiling, we identified a nine-gene expression signature, termed RESIST-M, which effectively stratified patients with the CMS4 subtype as well as those in a high-risk iCMS3-fibrotic subtype, both associated with the poorest overall survival. Notably, we observed that the RESIST-M signature demonstrated superior specificity in distinguishing CMS4 tumors and hence predict patient survival better than previously reported oxaliplatin-resistance signatures. Furthermore, we uncovered distinct therapeutic vulnerabilities in oxaliplatin-resistant CRC cells, highlighting new avenues for interventions. Collectively, our findings elucidate promising strategies aimed at mitigating metastatic progression and improving survival in patients with oxaliplatin-resistant CRC, particularly within the CMS4/iCMS3-fibrotic subtypes.

## Results

### Clinically relevant dosing with oxaliplatin drives emergence of drug-resistant, metastatic phenotype in CRC cell lines

**Fig. 1:**
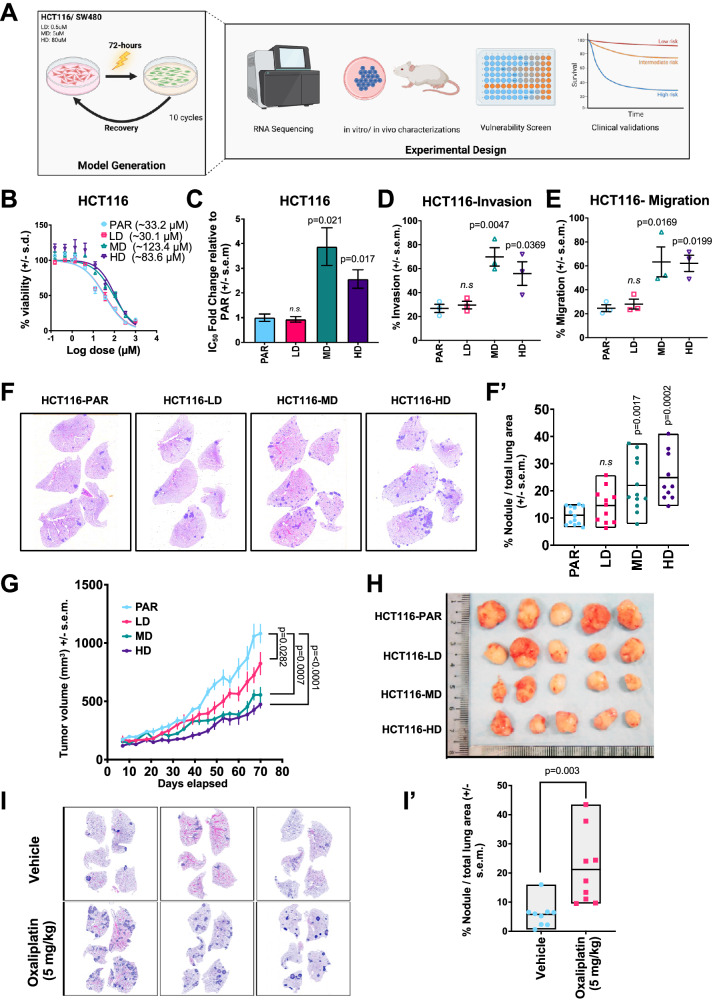
Clinically relevant dosing with oxaliplatin drives emergence of drug-resistant, metastatic phenotypes in CRC cell lines. **A** Schematic depicting generation of oxaliplatin-resistant models. HCT116 and SW480 CRC cells were treated with 0.5 µM (LD), 5 µM (MD), or 80 µM (HD) of oxaliplatin for 72 h and then left to recover in drug-free medium until confluent. Cells were then replated and treated again with oxaliplatin for 72 h. This dosing regime was repeated for 10 cycles. Oxaliplatin-resistant models were subjected to downstream transcriptomic and phenotypic characterizations. **B** Representative dose-response curves of oxaliplatin-resistant HCT116 models compared to parental cells (PAR). **C** Oxaliplatin IC_50_ fold change in HCT116-LD, MD, and HD relative to PAR. **D** Transwell invasion assay in HCT116-PAR, LD, MD, and HD. **E** Transwell migration assay in HCT116-PAR, LD, MD, and HD. **F** Representative H&E staining for spontaneous metastasis in lung sections from mice harboring xenografts of HCT116-PAR, LD, MD, and HD. **F’** Quantification of total tumor nodules area in lung sections of mice harboring xenografts of HCT116 parental and oxaliplatin-resistant models (*n* = at least 8). **G** In vivo tumor growth kinetics of HCT116-PAR, LD, MD, and HD xenografts (*n* = 5). **H** Corresponding image of HCT116-PAR, LD, MD, and HD xenografts at day 70. **I** Three representative H&E staining of lung sections in mice harboring HCT116-PAR xenografts treated with either vehicle or oxaliplatin (5 mg/kg). **I’** Quantification of tumor nodules area in lung sections from mice harboring HCT116-PAR xenografts treated with vehicle or oxaliplatin (*n* = 9). Statistical significance was determined using Ordinary one-way ANOVA followed by Dunnett’s multiple comparisons test for (**C**, **D**, **E**, **F’**). Ordinary two-way ANOVA followed by Dunnett’s multiple comparisons test was used for **G** and two-tailed unpaired *t*-test for **I’**. Figure 1A was created with BioRender.com.

Previous studies exploring chemoresistance and metastasis have frequently relied on models involving prolonged or supra-physiological drug exposure, which fail to reflect clinically relevant treatment conditions [12, 13]. To better mimic therapeutic settings, we investigated cellular adaptations under clinically relevant oxaliplatin exposure. Two colorectal cancer (CRC) cell lines, HCT116 and SW480, were subjected to 10 cycles of oxaliplatin treatment at three different concentrations: 0.5 µM (**L**ow-**D**ose; [HCT116-LD or SW480-LD]), 5 µM (**M**id-**D**ose; [HCT116-MD or SW480-MD]) and 80 µM (**H**igh-**D**ose; [HCT116-HD or SW480-HD]) (Fig. 1A), corresponding to 0.1x, 1x and 16x the maximum plasma concentration (C_max_) observed in patients [14].

Following treatment, HCT116 cells exposed to mid and high doses (5 µM and 80 µM, respectively) developed increased resistance to oxaliplatin (Fig. 1B, C and Supplementary Fig. S1A, B), while low dose treatment (0.5 µM) had no appreciable effect. Notably, HCT116-HD cells also acquired a “spindle-like” morphology not observed in other groups (Supplementary Fig. S1C), suggesting an epithelial-to-mesenchymal transition (EMT)-like phenotype. Indeed, transwell invasion and migration assays showed that oxaliplatin-treated HCT116-MD and HD, but not SW480-MD/HD, acquired enhanced invasive and migratory capabilities (Fig. 1D, E and Supplementary Fig. S1D, E). These findings were further supported in vivo, where HCT116-MD and HD xenografts displayed enhanced spontaneous lung metastases in immunodeficient mice (Fig. 1F, F**’**), a phenotype not observed in SW480 xenograft models (Supplementary Fig. S1F, G), mirroring the in vitro results. Interestingly, the oxaliplatin-resistant models also showed significantly reduced proliferation in vitro, along with markedly slower tumor growth compared to their respective parental lines (Fig. 1G, H and Supplementary Fig. S1H–K), suggesting that the increased metastatic capacity is independent of the growth or proliferative capacity of the CRC tumor cells.

To determine whether oxaliplatin could directly promote a more aggressive phenotype in vivo, and to better simulate the clinical context of neoadjuvant therapy, we administered five cycles of oxaliplatin (5 mg/kg) to mice bearing parental HCT116 or SW480 tumors. Consistent with our previous findings, oxaliplatin-treated HCT116 xenografts exhibited increased incidences of pulmonary metastasis (Fig. 1I, I**’**), a response not observed in mice with SW480 tumors (Supplementary Fig. 1L, M). Given the pronounced metastatic propensity of drug-resistant HCT116 cells, we selected this model for deeper investigation into the mechanisms underlying therapy-induced metastatic progression.

### Dysregulated expression of *SERPINE1* (PAI-1) promotes chemoresistance and metastasis in oxaliplatin-treated HCT116

**Fig. 2:**
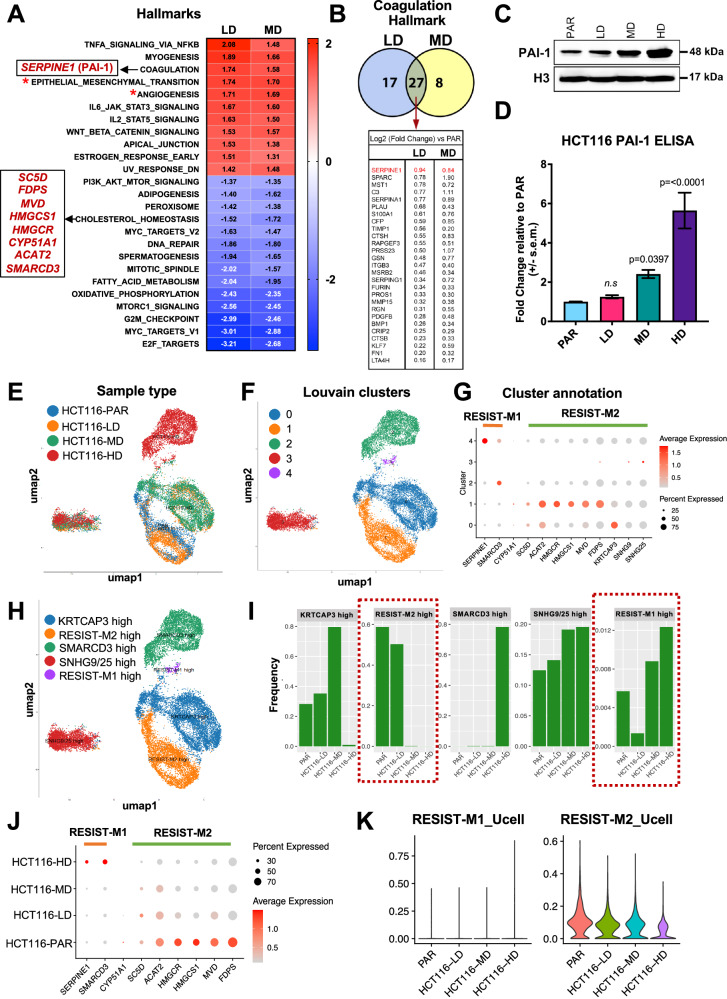
Dysregulated expression of *SERPINE1* (PAI-1) promotes chemoresistance and metastasis in oxaliplatin-treated HCT116 cells. **A** Significantly enriched hallmarks in HCT116-LD and MD using Gene Set Enrichment Analysis (GSEA) on differentially expressed genes obtained from bulk transcriptomic data of HCT116 resistant models. Red and blue indicates positively and negatively regulated genes, respectively, with the values of normalized enrichment score (NES) as indicated. Pathways with annotated with * denotes hallmarks commonly associated with drug-resistance and metastasis. Pathways and genes explored in this manuscript are highlighted black boxes. **B** Venn diagram of overlapping and exclusive genes from the leading edge of the coagulation hallmark in HCT116-LD and MD (top). Overlapping genes shared between HCT116-LD and MD from the leading edge of the coagulation hallmark (bottom). **C** Immunoblotting of PAI-1 in HCT116 PAR, LD, MD, and HD. **D** Fold change of PAI-1 protein in cell culture supernatant of HCT116 LD, MD, and HD relative to PAR determined using ELISA. **E** UMAP projection and sample type annotation of scRNA-seq data from the four scRNA-seq libraries: HCT116-PAR/LD/MD/HD. **F** UMAP projection and Louvain-based clustering of the four samples identifies 5 distinct cell states (cl 0-5) defining the parental and resistant conditions. **G** Dot plot showing differentially expressed genes (DEGs) of the 5 identified clusters (cl 0-5). RESIST-M gene signature, wherein RESIST-M1 genes denote *SERPINE1*, *SMARCD3*, and RESIST-M2 genes denote *SC5D, FDPS, MVD, HMGCS1, HMGCR, CYP51A1, ACAT2* can distinguish the cell states defined by clusters 1, 2, 4. **H** UMAP projection and annotation of the 5 identified cell states based on DEGs. **I** Proportions of the different annotated clusters across the sample type shows loss and gain in clusters with high RESIST-M2 and high RESIST-M1 signature, respectively. **J** Dot plot showing differential expression of RESIST-M genes as they increase and decrease, respectively, depending on the dose of oxaliplatin and type of metastatic phenotype acquired. **K** Violin plot showing Ucell score for RESIST-M1 and M2 genes, increasing and decreasing, respectively, as the selection pressure of oxaliplatin is increased. Statistical significance was determined using Ordinary one-way ANOVA followed by Dunnett’s multiple comparisons test for (**D**).

To uncover potential molecular mechanisms driving the observed drug-resistant and metastatic phenotypes, we performed bulk RNA sequencing on oxaliplatin-treated and untreated cells. Gene Set Enrichment Analyses (GSEA) revealed upregulation of key pathways linked to metastasis, such as EMT and angiogenesis, in HCT116-LD and MD cells (Fig. 2A, denoted with *; Supplementary Fig. S2A), but not in SW480 cell lines (Supplementary Fig. S2B). Interestingly, HCT116-LD and MD cells showed remarkably similar enriched hallmarks (Fig. 2A), whereas gene sets enriched in HCT116-HD cells, exposed to the supra-physiological dose of 80 µM, were distinctly different (Supplementary Fig. S2A). This suggests that while similar phenotypes (i.e., drug resistance and increased metastasis) emerge across treatment conditions, high-dose oxaliplatin may select for cell-state-specific biological programs that support the survival of pro-metastatic, drug-tolerant persister (DTP) cells [15].

Amongst the pathways enriched in HCT116-LD and MD cells, the coagulation hallmark emerged as significantly upregulated (Fig. 2A). While coagulation pathway has been implicated in tumor progression and metastatic spread [16, 17], specific molecular mechanisms remain relatively understudied. Comparative analysis of differentially expressed genes within the coagulation pathway between HCT116-LD and MD revealed *SERPINE1* as the top upregulated and potentially druggable candidate (Fig. 2B). *SERPINE1*, which encodes the plasminogen activator inhibitor type 1 protein (PAI-1), is a critical homeostatic regulator of fibrinolysis and thrombosis [18], and has previously been associated with both drug resistance and metastasis in various cancer types [19, 20]. Notably, ELISA and immunoblot analyses showed increased PAI-1 expression specifically in HCT116-MD and HD cells, but not in HCT116-LD (Fig. 2C, D), suggesting its potential involvement in mediating acquired resistance and metastatic phenotypes.

To gain deeper insights into therapy-induced acquired resistance and metastatic phenotypes, we performed single-cell RNA-sequencing (scRNA-seq) on both the parental and oxaliplatin-resistant cell lines. Louvain-based clustering revealed five distinct transcriptomic cell states (Fig. 2E–I). Amongst them, cluster 3 was unique for its loss of lineage-defining markers, with a subset of cells expressing *SNHG* (referred to as *SNHG9/25* high, *cl 3*). Notably, we found *cl 3* to be present in both parental and resistant cell lines (Fig. 2E–I and Supplementary Fig. S2C), suggesting the presence of pre-existing drug-tolerant cell states that become enriched (Darwinian selection) upon oxaliplatin treatment. Additionally, leveraging on the differentially-expressed genes identified from bulk-RNA seq of parental and resistant cell lines, we uncovered two key gene expression signatures, RESIST-M1 (where RESIST-M denotes **RESI**stance-**I**nduced **S**igna**T**ure for CRC **M**etastasis) characterized by high expression of *SERPINE1* and *SMARCD3*; and RESIST-M2, defined by genes involved in cholesterol biosynthesis (*SC5D, FDPS, MVD, HMGCS1, HMGCR, CYP51A1, ACAT2*). These RESIST-M signatures mapped to two distinct single cell clusters: RESIST-M1 to cluster 4 and RESIST-M2 to cluster 1 (Fig. 2G). As resistance and metastatic traits increased, we observed a progressive gain of the RESIST-M1 signature, including elevated SERPINE1 and SMARCD3, alongside a loss of RESIST-M2 expression (Fig. 2H–K and Supplementary Fig. S2D, E). Intriguingly, HCT116-HD displayed a divergent evolutionary trajectory. These cells exhibited high *SMARCD3* expression, a concomitant loss of *KRTCAP3* expression, and the loss of heterogeneity, as defined by the predominance of a single cluster, *cl 2* (Fig. 2I, SMARCD3 high panel). This loss of heterogeneity and clonal dominance observed in cells selected with high-dose oxaliplatin was accompanied by increased differentiation scores, as revealed by CytoTRACE analysis (Supplementary Fig. S2F–H). Pseudotime analyses, excluding *SNHG9/25* high cluster, indeed demonstrated that HCT116-HD cell lines emerge as a distinct drug-tolerant cell population (Supplementary Fig. S2I, J). In contrast, cell populations treated with lower and more clinically relevant concentrations (LD and MD) revealed a continuum of cell-states that partly retained transcriptomic heterogeneity found in the parental, untreated cells (Fig. 2E–H and Supplementary Fig. S2I, J). Taken together, these findings indicate therapy-induced resistance arises through both selection of pre-existing subpopulations (defined by their heterogeneous cell states) and adaptive transcriptional reprogramming, with distinct molecular trajectories emerging in response to varying therapeutic dosing schedules.

### Genetic and pharmacological inhibition of *SERPINE1* (PAI-1) reverses chemoresistance and metastasis in oxaliplatin-treated HCT116

**Fig. 3:**
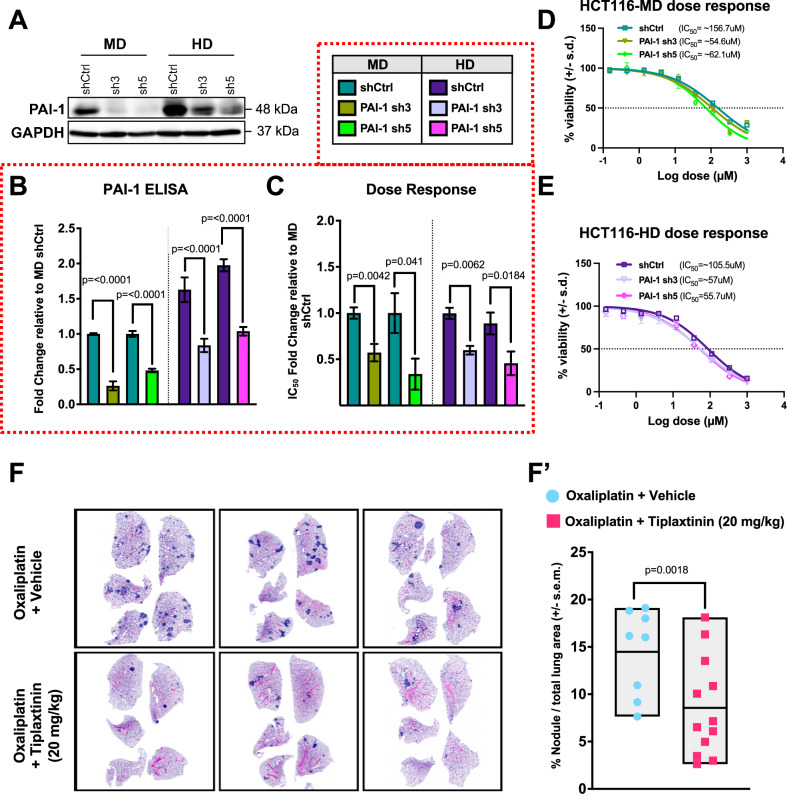
Genetic and pharmacological inhibition of *SERPINE1* (PAI-1) reverses chemoresistance and metastasis in oxaliplatin-resistant HCT116 cells. **A** Immunoblotting of PAI-1 in HCT116-MD and HD knocked down for the protein using two unique shRNAs (sh3 and sh5). **B** Fold change of PAI-1 protein in cell culture supernatant of HCT116-MD and HD knocked down for the protein relative to MD shRNA control (shCtrl). **C** Oxaliplatin IC_50_ fold change HCT116-MD and HD knocked down for the protein relative to MD shCtrl. Representative dose response curves of HCT116-MD shCtrl, PAI-1 sh3 and sh5 (**D**) and HCT116-HD shCtrl, PAI-1 sh3 and sh5 (**E**). **F** Three representative H&E staining for spontaneous metastasis in lung sections of mice harboring HCT116-PAR xenografts treated with oxaliplatin alone, or in combination with PAI-1 inhibitor, tiplaxtinin (20 mg/kg). **F’** Quantification of tumor nodules area in lung sections of mice harboring HCT116-PAR xenografts treated with oxaliplatin alone, or in combination with tiplaxtinin (20 mg/kg). Statistical significance was determined using Ordinary one-way ANOVA followed by Sidak’s multiple comparisons test for (**B**, **C**) and two-tailed unpaired *t*-test for (**F’**).

To investigate the functional contribution of PAI-1 to oxaliplatin resistance, we conducted loss-of-function studies using two distinct shRNAs targeting the *SERPINE1* gene in the oxaliplatin-resistant HCT116 cells. This resulted in a robust knockdown of the protein as validated by immunoblotting and ELISA (Fig. 3A, B). Notably, shRNA-mediated silencing of PAI-1 re-sensitized both HCT116-MD and HCT116-HD to oxaliplatin treatment (Fig. 3C–E). Importantly, pharmacological inhibition of PAI-1 using tiplaxtinin [21] significantly reduced the incidence of spontaneous pulmonary metastases in oxaliplatin-treated HCT116 xenograft models (Fig. 3F, F**’**). Collectively, our findings reveal a previously unrecognized role of oxaliplatin-induced *SERPINE1* in promoting both chemoresistance and metastasis in CRC.

### Elevated *SERPINE1* (PAI-1) expression is linked to dysregulated cholesterol-TGF-β signaling and increased chromatin accessibility

**Fig. 4:**
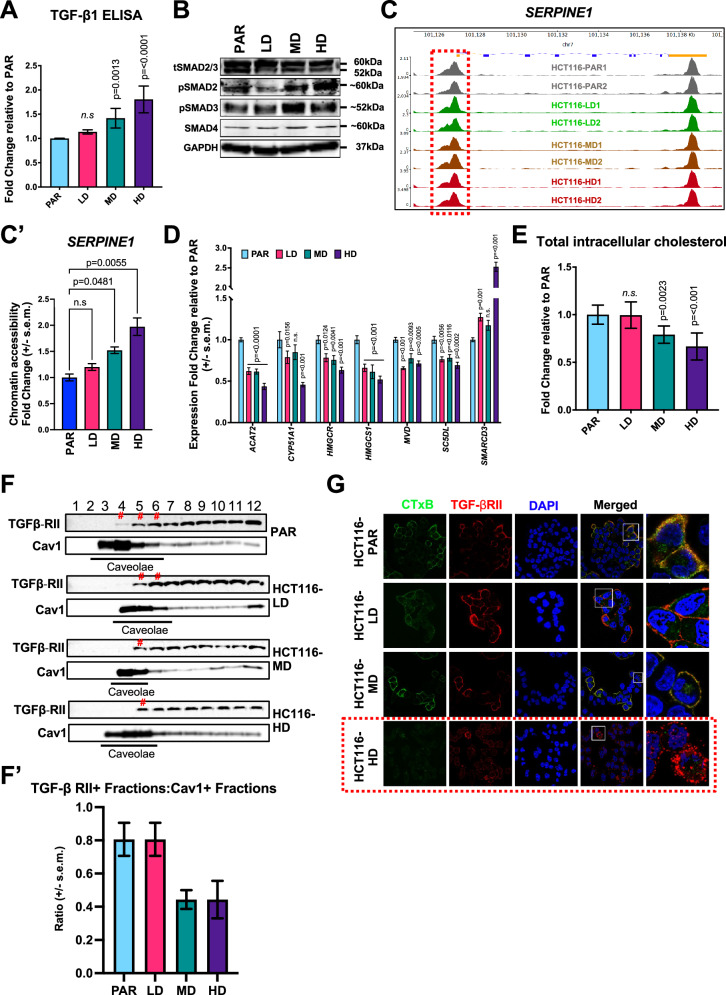
Enhanced expression of *SERPINE1* (PAI-1) is associated with dysregulated cholesterol-TGF-β signaling and increased chromatin accessibility. **A** Fold change of TGF-β1 protein in cell culture supernatant of HCT116 LD, MD and HD relative to PAR determined using ELISA. **B** Immunoblotting of total SMAD2/3 (tSMAD2/3), phospho-SMAD2 (pSMAD2), phospho-SMAD3 (pSMAD3) and SMAD4 in oxaliplatin-resistant HCT116 models. **C**, ATAC-seq signal tracks near the *SERPINE1* gene. Red dotted box indicates area of differential peaks near 5′ UTR quantified for mean signal intensity. **C’** Normalized mean signal intensity denoted as chromatin accessibility fold change in *SERPINE1* promoter regions across oxaliplatin-resistant HCT116 models relative to PAR. **D** Expression fold change of cholesterol-related genes identified in Fig. 2A in the resistant models relative to parental cells determined using quantitative real-time PCR. **E** Fold change of total intracellular cholesterol in resistant models relative to parental cells determined using ELISA. **F** Representative immunoblots of TGFBRII and caveolin after fractionation. # indicates TGFBRII found in lipid raft (caveolin-1 [Cav1] high/positive) fractions. **F’** Quantification of three independent immunoblots denoting the ratio of TGFBRII-positive fractions to Cav1 positive fractions. **G** Immunofluorescence staining for colocalization of cholera toxin subunit B (CTxB; green, lipid rafts) and TGF-β receptor II (TGF-βRII; red). White box denotes selected regions that are magnified. Red dotted box highlights the high oxaliplatin dose (HCT116 HD) that show most drug-tolerant persistent cells with reduced lipid rafts colocalized with TGF-βRII. Statistical significance was determined using Ordinary one-way ANOVA followed by Dunnett’s multiple comparisons test for (**A**, **C’**, **E**), and Ordinary 2-way ANOVA followed by Dunnett’s multiple comparisons test for **D**.

Given the pivotal role of *SERPINE1* in promoting metastasis and oxaliplatin resistance, we examined the molecular mechanisms responsible for its upregulation following oxaliplatin treatment. *SERPINE1* is a known transcriptionally target of TGF-β signaling [22]. ELISA and immunoblot analysis of key TGF-β signaling pathway proteins revealed elevated levels of the TGF-β cytokine (Fig. 4A) and enhanced canonical SMAD signaling, evidenced by increased phosphorylation of SMAD2/3, in HCT116-MD and HD cells (Fig. 4B). Furthermore, ATAC-seq analysis showed increased chromatin accessibility at the SERPINE1 5’ UTR region, specifically at the region containing a consensus SMAD-binding motif (Fig. 4C, C**’** and Supplementary Fig. S3A, B), suggesting that enhanced TGF-β signaling and epigenetic remodeling contribute to SERPINE1 overexpression in resistant cells.

TGF-β signaling is also tightly regulated by receptor trafficking and localization, particularly through cholesterol-rich lipid raft microdomains [23]. Notably, GSEA analysis of RNA-seq data from oxaliplatin-treated HCT116 cells revealed significant downregulation of genes involved in cholesterol biosynthesis (Fig. 2A). Specifically, we identified eight cholesterol-related genes whose expression was significantly reduced (Supplementary Fig. S4A) and validated seven of them using qPCR (Fig. 4D). Direct measurement of intracellular cholesterol confirmed a marked reduction of the sterol content in HCT116-MD and HD cells (Fig. 4E).

Given cholesterol’s critical role in maintaining lipid-raft integrity and regulating various signaling cascades by controlling receptor localization and trafficking between raft and non-raft regions [24], we hypothesized that reduced cholesterol levels could alter TGF-β receptor distribution across membrane compartments to modulate TGF-β activity in HCT116-MD and HD cells. Using sucrose gradient density fractionation, we found a notable reduction of TGF-β receptor 2 (TGFβ-RII) within lipid raft domains (caveolin-1 positive fractions; Cav1) in resistant cells (Fig. 4F, F**’**). Consistently, immunofluorescence staining corroborated these findings, showing decreased colocalization of the receptors in lipid raft regions (cholera-toxin subunit B positive staining; CTxB) of the HCT116-MD and HD cells (Fig. 4G). These findings suggest that decreased cholesterol levels diminish the lipid raft localization of TGFBRII.

As lipid raft-associated TGF-β receptors are known to be more susceptible to ubiquitination and subsequent degradation [25], their redistribution may indicate reduced turnover and thereby enhanced TGF-β signaling. This increased signaling likely contributes to sustained SMAD2/3 activity and subsequent upregulation of SERPINE1 in oxaliplatin-resistant cells.

### RESIST-M gene signature derived from oxaliplatin-resistant models is preferentially enriched in the CMS4 subtype of CRC patients

Overexpression of *SERPINE1* has been linked to adverse clinical outcomes in various cancer types [26, 27]. Consistent with this, we found that *SERPINE1* upregulation was associated with enhanced invasive disease and poorer prognosis across multiple CRC patient cohorts (Supplementary Fig. S4B–G). In this study, we show that not only *SERPINE1*, but also high expression of RESIST-M1 (*SERPINE1*, *SMARCD3*) and low expression of RESIST-M2 (*SC5D, FDPS, MVD, HMGCS1, HMGCR, CYP51A1, ACAT2*) genes were significantly enriched in the CMS4 subtype of CRC, which is strongly associated with poor prognosis and aggressive disease.

**Fig. 5:**
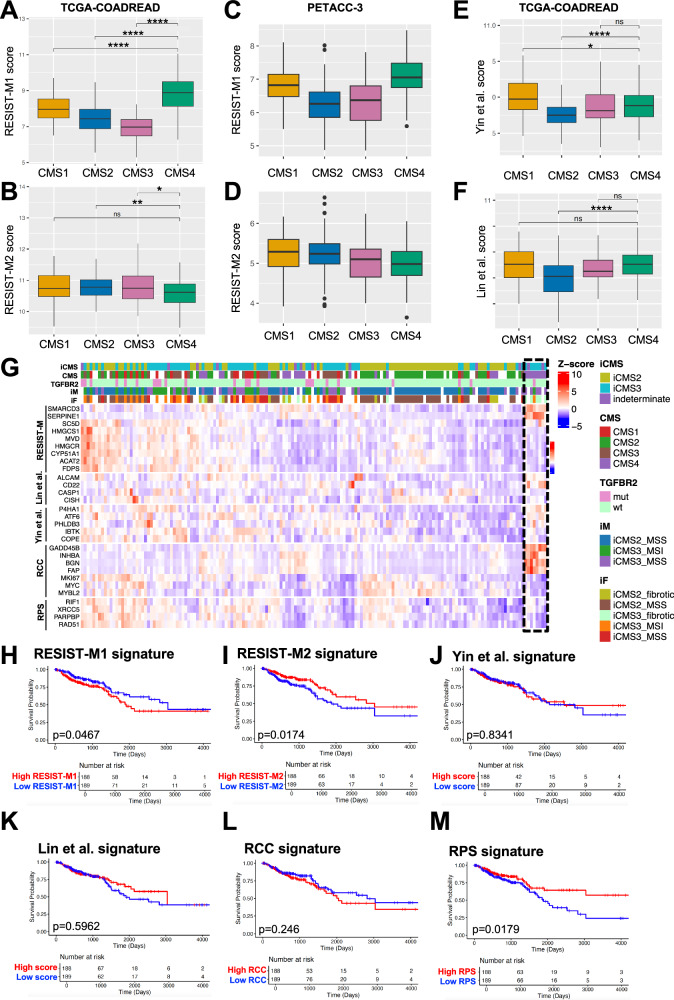
RESIST-M gene signature derived from oxaliplatin-resistant models is specifically enriched in the CMS4 subtype of colorectal cancer patients. Expression of RESIST-M1 and M2 genes in TCGA-COADREAD (*n* = 377) (**A**, **B**) and PETACC-3 dataset (*n* = 604) (**C**, **D**). Mean expression of gene signatures reported for oxaliplatin resistance from Yin et al. **E** and Lin et al. **F** in TCGA-COADREAD dataset. **G** Heatmap showing publicly available bulk RNA-seq data from Singapore colorectal cancer patients (*n* = 162, SG-BULK, ID: syn26720761) with RESIST-M signature. Patients with high expression of both *SERPINE1* and *SMARCD3* and low expression of *SC5D, FDPS, MVD, HMGCS1, HMGCR, CYP51A1*, and *ACAT2* are enriched in patients of a CMS4/iCMS3-fibrotic subtype. Each column denotes data on a single CRC patient, and each row indicates the patient subtype based on available clinical data and z-scored values based on RESIST-M, Lin et al., Yin et al., RCC, RPS gene expression from top to bottom. Seven of the twelve genes from the RCC gene were used in this analysis which includes stromal genes (*INHBA, BGN, FAP)*, cell cycle genes *(MKI67, MYC, MYBL2)* and *GADD45B*. RPS gene score includes mean expression of four DNA repair genes- *RIF1, XRCC5, PARPBP*, and *RAD51*. Clinical data shown in the legend include patient subtype based on either iCMS, CMS, TGFBR2 mutation, iCMS-microsatellite (iM), or iCMS-fibrosis status (iF). Kaplan Meier curves for overall survival of CRC patients in TCGA COADREAD dataset stratified using RESIST-M1 (**H**), RESIST-M2 (**I**), Yin et al. (**J**), Lin et al. (**K**), RCC (**L**), RPS (**M**) gene signatures. Statistical significance for **A**–**F** was determined using Wilcoxon rank-sum test, and log-rank p test was done for **H**–**M**. * represents a *p* value of <0.05, ** represents <0.01, **** represents <0.001.

Molecular stratification studies, including Consensus Molecular Subtypes (CMS) [28] and iCMS subtypes [29], have shown that patients with CMS4 and iCMS3-fibrotic subtypes derive minimal benefit from oxaliplatin treatment and typically experience the worst relapse-free and overall survival [5, 29]. To evaluate the clinical relevance of the RESIST-M signatures, we analyzed two CRC datasets: (a) TCGA COADREAD (*n* = 377, bulk RNA-seq of CRC patients with CMS annotations) (Fig. 5A, B), and (b) PETACC-3 dataset [30] (*n* = 604, bulk RNA-seq of CRC patients treated with 5FU-based chemotherapy) (Fig. 5C, D). We found that RESIST-M1 genes were significantly upregulated, while RESIST-M2 genes were downregulated in CMS4 patients. This pattern was not observed with other previously published oxaliplatin resistance signatures, such as those by Yin et al. [31] and Lin et al. [32], which failed to differentiate between the different CMS subtypes (Fig. 5E, F). This discrepancy may reflect the fact that these signatures (Yin et al., Lin et al.) were derived from computational analysis of clinical datasets that do not display clear evidence of an oxaliplatin resistance-induced metastatic phenotype.

Strikingly, we observed that the RESIST-M genes (high RESIST-M1 and low RESIST-M2) could specifically stratify a subset of patients that were of CMS4 and iCMS3-fibrotic subtype in the SG-BULK dataset [29] (synapse accession code: syn26720761) (Fig. 5G, **Black dotted box**). In contrast, other predictive models, including the Yin et al., Lin et al., colon cancer recurrence score (denoted as RCC) [33], and Recombination Proficiency Score (RPS) [34] signatures, all failed to identify this high-risk subgroup of patients (Fig. 5G). RCC and RPS scores are frequently used in cancers to predict recurrence and DNA mismatch repair, respectively. The RCC score includes a panel of 12 genes, with 7 cancer-related genes (stromal genes: *INHBA, BGN*, and cell cycle genes: *MKI67, MYC, MYBL2*, *GADD45B*) and 5 reference genes, and is used to predict CRC recurrence. However, the RCC score does not predict the relative benefit from oxaliplatin treatment [33]. Importantly, our analysis showed that patients with RESIST-M signature exhibited high expression of stromal genes like *INHBA, BGN, FAP* and high *GADD45B*, while having low expression of cell cycle related genes like *MKI67, MYBL2, MYC*, further supporting their classification as chemoresistant.

Another clinical marker, *CDX2*, has been implicated in chemotherapy responsiveness, with *CDX2-*negative CRC patients reportedly benefiting from chemotherapy, but not specifically from oxaliplatin [35]. With our models, we provide evidence that oxaliplatin-resistant cancer cells have low *CDX2* expression; display lower expression of both RPS and RCC genes, and are characterized by RESIST-M signature, all of which collectively correlate with clinical resistance to oxaliplatin (Supplementary Fig. S6H, I).

Importantly, the RESIST-M signature outperformed previous gene sets in predicting poor prognosis across several independent datasets. For instance, patients with high RESIST-M1 and low RESIST-M2 expression had significantly worse outcomes in the TCGA COADREAD cohort (Fig. 5H, I and Supplementary Fig. S5A), three additional public datasets (Supplementary Fig. S4B, C), and the PETACC-3 cohort, which included patients who received adjuvant chemotherapy (Supplementary Fig. S5D–F). Particularly, low RESIST-M2 expression was a strong predictor of poor relapse-free survival within CMS4 patients in the PETACC-3 cohort. Collectively, these findings highlight the RESIST-M signature as a robust and clinically relevant biomarker for identifying oxaliplatin-resistant, pro-metastatic CRC, particularly within the CMS4 and iCMS3-fibrotic subtypes, and may offer a valuable tool prognostication and therapeutic stratification.

### Oxaliplatin-resistant HCT116 cells display enhanced therapeutic vulnerability against drugs targeting cholesterol biosynthesis and ROS

**Fig. 6:**
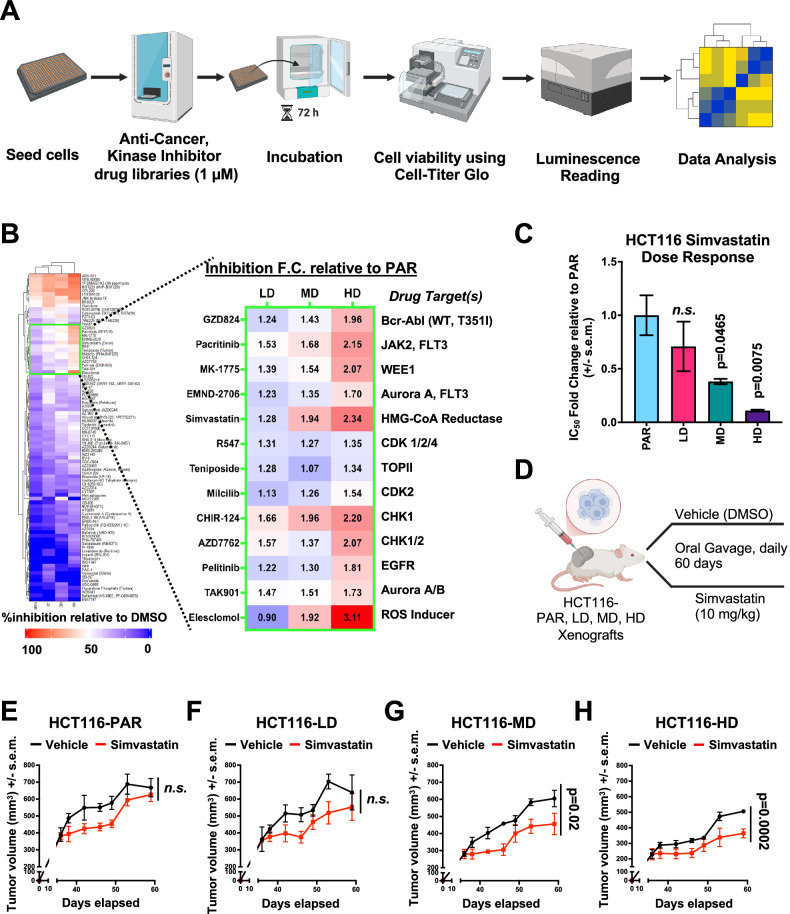
Oxaliplatin-resistant HCT116 cells exhibit increased sensitivity to inhibitors of cholesterol biosynthesis and oxidative stress pathways. **A** Workflow for synthetic lethal drug screen. Cells were seeded and incubated before drugs from the anti-cancer and kinase inhibitor libraries were added to the plate for a 72-h treatment. Cell viability was then determined by adding Cell-Titer Glo, and luminescence intensity was determined using a plate reader for further data analysis. **B** Heatmap of drug inhibition on cell viability relative to DMSO control of HCT116 oxaliplatin-resistant models using a library of FDA-approved anti-cancer and kinase inhibitor libraries (left panel). Red indicates a 100% inhibition of cell viability, while blue indicates no inhibition of cell viability compared to DMSO control. Drugs that displayed differential sensitivity between HCT116-PAR and oxaliplatin-resistant models are boxed in green along with inhibition fold change relative to HCT116-PAR (right panel). **C** Simvastatin IC_50_ fold change in HCT116- LD, MD, and HD relative to PAR. **D** Schematic depicting in vivo treatment of simvastatin in mice harboring xenografts of HCT116-PAR, LD, MD, and HD. Tumor growth curves of simvastatin-treated HCT116 PAR (**E**), LD (**F**), MD (**G**), and HD (**H**) relative to drug vehicle treatment. Statistical significance for **D**–**G** was determined using Ordinary one-way ANOVA followed by Dunnett’s multiple comparisons test. Figure 6A, D were created with BioRender.com.

Resistance to one therapeutic agent is often accompanied by acquiring increased sensitivity to another [36]. To uncover such potential vulnerabilities in our oxaliplatin-resistant CRC models, we conducted a synthetic lethal drug screen using a panel of FDA-approved anticancer and kinase inhibitor libraries (Fig. 6A–H for HCT116, Supplementary Fig. S6A, B for SW480). Notably, oxaliplatin-resistant HCT116 cells exhibited increased sensitivity to simvastatin, an inhibitor of HMG-CoA, that impairs cholesterol biogenesis (Fig. 6B). Dose response experiments in vitro further confirmed a significant increase in sensitivity towards simvastatin in both HCT116-MD and HD cells (Fig. 6C). To see whether this sensitivity translated in vivo, we treated tumor bearing mice with simvastatin (Fig. 6D). Consistent with our in vitro observations, simvastatin treatment led to a marked reduction in tumor growth, but only in mice harboring HCT116-MD and HD xenografts (Fig. 6E–H). These results support the concept that further inhibition of cholesterol biosynthesis, which is already impaired in oxaliplatin-resistant cells, can severely compromise cell viability. This is consistent with prior studies demonstrating that targeting cholesterol biogenesis can be exploited to inhibit breast cancer metastasis [37]. However, while there are limited reports on the role of statins in mitigating oxaliplatin-induced neurotoxicity [38], their therapeutic potential in combating oxaliplatin-induced metastasis remains largely unexplored.

In addition to simvastatin, our screen identified elesclomol-a copper chelator known to induce oxidative stress and ferroptosis in CRC [39], and inhibit CMS4 subtype-associated with peritoneal metastasis [40] as another promising candidate. The increased levels of reactive oxygen species (ROS) detected in oxaliplatin-resistant HCT116 cells (*data not shown*), as well as the CMS4-like metastatic properties of the resistant cell lines, may underlie their heightened sensitivity to ROS-inducing agents. In summary, our findings uncover key therapeutic vulnerabilities in oxaliplatin-resistant CRC models, highlighting potential strategies to overcome resistance. These insights offer new avenues for targeting oxaliplatin-resistant tumors, particularly the pro-metastatic CMS4 subtype of CRC marked by the RESIST-M gene expression.

## Discussion

Traditional methods of modeling chemoresistance and metastasis often rely on prolonged or supra-physiological drug exposure, which fails to accurately reflect the clinical treatment regimen [12, 13]. In this study, we established a more clinically-relevant model of oxaliplatin resistance using cyclical and physiological dosing in two CRC cell lines, HCT116 and SW480. This approach allowed us to capture cellular and molecular adaptations to treatment using a comprehensive array of methods, including bulk and single-cell transcriptomics, ELISA, western blotting, immunohistochemistry, and preclinical, in vivo murine tumor models.

Our results demonstrate that repeated exposure to clinically relevant mid and high doses of oxaliplatin induced drug resistance and metastatic traits in CRC cells. Notably, HCT116 cells developed a more pronounced metastatic phenotype than SW480, likely reflecting underlying genomic, transcriptomic, and epigenetic differences driving drug-resistance and metastasis. Transcriptomic analyses revealed that drug resistance in both lines was associated with activation of pro-inflammatory pathways such as JAK-STAT, NF-κB, and IFN-γ signaling. In HCT116 cells, oxaliplatin also induced epithelial-to-mesenchymal transition (EMT) programs, potentially contributing to their increased invasiveness, consistent with the pro-metastatic role of mesenchymal features [41].

Deep molecular and phenotypic characterization of the resistant models uncovered a shift towards pro-coagulation and metabolically-reprogrammed states, characterized by an upregulation in coagulation related pathways, and downregulation of cholesterol biosynthesis, oxidative phosphorylation (OXPHOS), and fatty-acid metabolism. Bulk and scRNA-seq analysis of oxaliplatin-resistant models revealed a significant upregulation of *SERPINE1*, a gene involved in the coagulation pathway, along with consistent suppression of key cholesterol-biosynthesis genes. These insights led to the development of the RESIST-M signature, comprising two gene sets: RESIST-M1 (e.g., *SERPINE1*, *SMARCD3*) and RESIST-M2 (e.g., *SC5D, FDPS, MVD, HMGCS1, HMGCR, CYP51A1, ACAT2*). Cells with high RESIST-M1 and low RESIST-M2 expression, particularly in HCT116 cells, exhibited both resistant and metastatic traits. Functional inhibition of *SERPINE1*, either using shRNA or pharmacological inhibitors like tiplaxtinin, resensitized resistant cells to oxaliplatin and reduced metastatic potential, establishing *SERPINE1* as a key player in oxaliplatin resistance-induced metastasis in CRC. Consistent with our findings, previous studies by independent groups have also demonstrated that *SERPINE1*, either tumor cell-intrinsic or cell-extrinsic, contributes to drug resistance and metastasis in various cancers, including breast [20] and head and neck cancers [19]. To the best of our knowledge, this study is the first to link *SERPINE1* with oxaliplatin resistance and metastasis in CRC.

Mechanistically, our data suggest that oxaliplatin-resistant cells co-opt both *SERPINE1* and altered cholesterol homeostasis to support survival and metastatic progression. Alterations in cellular cholesterol can disrupt the structure and composition of plasma membranes, particularly the caveolin-coated lipid rafts, which negatively regulate signaling pathways such as TGF-β^22^. In our models, cholesterol depletion disrupted TGF-β receptor localization within lipid rafts, leading to enhanced canonical TGF-β signaling. This, in turn, upregulated *SERPINE1* and EMT programs, thereby linking altered lipid metabolism to chemoresistance and metastatic capacity. These observations contrast with cancer types in which elevated cholesterol biosynthesis promotes tumor growth [42], highlighting a distinct vulnerability in oxaliplatin-resistant CRC. It remains to be elucidated why and how cholesterol biosynthesis is being downregulated in oxaliplatin-resistant CRC cells.

The clinical utility of this study is underscored by the validation of the RESIST-M signature in multiple independent CRC patient cohorts. RESIST-M1 and RESIST-M2 gene signatures were typically anti-correlated and strongly enriched in CMS4-CRC, a subtype characterized by aggressive, stromal-rich tumors with poor prognosis and poor response to oxaliplatin. Intriguingly, RESIST-M signature was particularly enriched in the CMS4 and iCMS3-fibrotic subtype within SG-BULK dataset, further emphasizing its association with pro-metastatic disease. Indeed, these subtypes have consistently shown high metastatic potential and poor response to oxaliplatin in clinical trials, such as NSABP C-07 and MOSAIC [3, 4].

Finally, synthetic lethal screens using drug libraries revealed that RESIST-M positive oxaliplatin-resistant HCT116 models were particularly sensitive to simvastatin, a cholesterol-lowering drug, and elesclomol, an inducer of reactive oxygen species (ROS), known to inhibit CMS4 CRC associated with peritoneal metastasis, via copper-dependent ferroptosis [39, 40]. These findings offer compelling therapeutic alternatives for targeting oxaliplatin-resistant, pro-metastatic CRC. In particular, our data support combining cholesterol biosynthesis inhibitors or ROS-inducing agents with standard chemotherapies as a strategy to overcome resistance and impede metastatic progression.

**Fig. 7:**
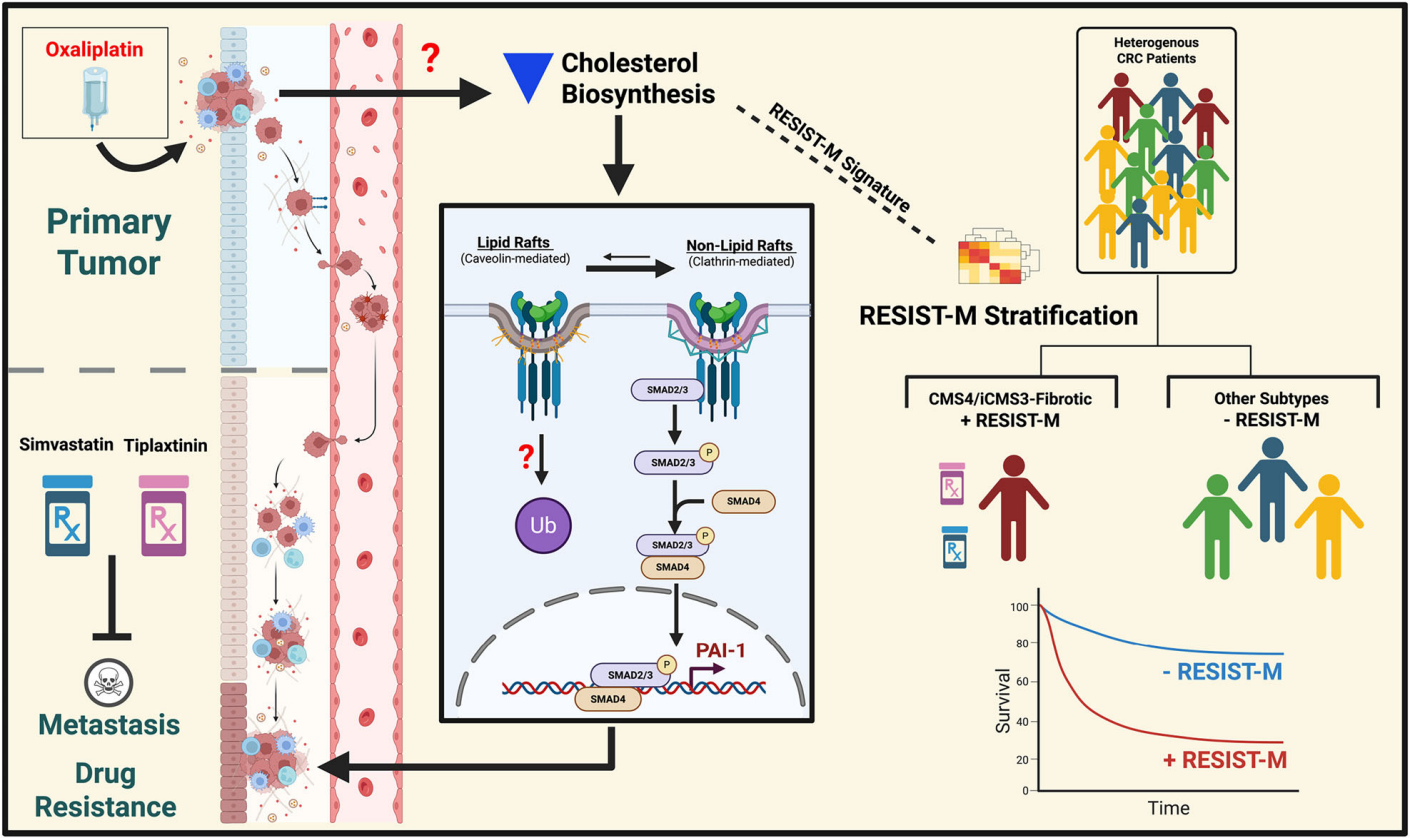
Oxaliplatin-resistant models reveal gene signatures associated with, and strategies to overcome drug resistance-induced metastasis. Oxaliplatin-resistant colorectal cancer (CRC) cells downregulate cholesterol biosynthesis, disrupting the localization of TGF-β receptors within caveolin-positive lipid rafts, which enhances TGF-β signaling, leading to upregulation of *SERPINE1*, a key driver of metastatic and invasive phenotypes in resistant CRC cells. A newly characterized SERPINE1-associated transcriptomic signature, RESIST-M, effectively stratifies CRC patients into the CMS4/iCMS3-fibrotic subtype—linked to poor prognosis, metastatic relapse, and oxaliplatin resistance. Pharmacological inhibition of PAI-1 or depletion of cellular cholesterol using statins reduces metastatic colonization and re-sensitizes resistant CRC cells to oxaliplatin. Created with BioRender.com.

In conclusion, this study presents a clinically relevant model of oxaliplatin resistance that provides key mechanistic insights and identifies actionable therapeutic targets that can be harnessed in the future to reverse therapy resistance and inhibit metastasis. We provide three key contributions: (i) a refined model for studying resistance to first-line chemotherapy, such as oxaliplatin, in CRC; (ii) a novel RESIST-M gene signature predictive of resistance-induced metastasis; and (iii) promising therapeutic candidates for targeting CMS4/iCMS3-fibrotic-like CRC subtypes (Fig. 7). Our models represent aggressive clinical subtypes of CRC (CMS4/iCMS3-fibrotic), making them a valuable resource for future investigations into chemoresistance and metastasis. The RESIST-M signature, derived from functionally validated resistant models, demonstrates robust prognostic power, surpassing previously reported signatures in its ability to predict patient outcomes. Targeting key vulnerabilities in these models, including *SERPINE1* inhibitors and cholesterol metabolism with statins, may provide an effective strategy for overcoming oxaliplatin resistance in the clinic, ultimately improving outcomes for patients with metastatic CRC.

## Materials and methods

### Cell culture

All cell lines were grown in standard cell culture conditions (37 °C, 5% CO_2_). HCT116 and SW480 were obtained from ATCC and maintained in Modified McCoy’s 5A media containing L-Glutamine (Gibco) supplemented with 10% FBS, 1% penicillin/streptomycin, and 1% sodium pyruvate. All cell cultures were routinely tested for the presence of mycoplasma using the MycoAlert Mycoplasma Detection Kit (Lonza, USA) following manufacturer’s protocol.

### Generation of oxaliplatin-resistant cell lines

Oxaliplatin (CAS#: 61825-94-3, Tocris) was dissolved in DMSO. Cells were treated for 72 h with different concentrations of oxaliplatin- 0.5 µM, 5 µM, and 80 µM, before changing to drug-free media. The drug-free media was replaced every alternate day until cells became confluent. Cells were then sub-cultured once they reached confluency and treated at the same dose for 72 h. This treatment cycle was repeated for a total of 10 times. The resulting oxaliplatin-resistant cell lines were then used in subsequent experiments and routinely maintained in drug-free media.

### In vitro dose-response assay

Approximately 5000–8000 cells were seeded into each well of a 96-well plate and incubated for 48 h. Cells were then treated with oxaliplatin for 72 h at nine different doses serially diluted 3-fold with a starting dose of 1000 µM. DMSO was used as a no-drug control. Cell viability was measured using CellTiter-Glo (Promega) following manufacturer’s protocol. Data points were plotted in Prism 9 and analyzed using the nonlinear regression function “log(inhibitor) vs. normalized response—Variable slope”.

### Cell proliferation assays

Real-time proliferation kinetics was done using IncuCyte (Essen Bioscience) following manufacturer’s protocol. Briefly, 5000 cells were seeded and cultured in 96-well plates, and images were taken every hour for the next 72 h. Percentage cell confluence was quantified using the in-built IncuCyte software.

### Transwell invasion and migration assays

Cultrex 96 Well BME Cell Invasion Assay kits (RnD Systems, 3455-096-K) were used for transwell invasion and migration assays following manufacturer’s protocol. Briefly, cells seeded prior to assay and grown to about 70% confluency were treated with 10 μg/mL of Mitomycin C (CAS#: 50-07-7, MedKoo Biosciences) for 2 h. 5 × 105 cells resuspended in serum-free media were seeded into each upper chamber well in triplicate. Wells used for invasion assays were pre-coated overnight with 0.1X Matrigel (Corning). Uncoated wells were used for migration assays. Complete media was added to the lower chamber. Cells that have migrated or invaded to the bottom of the transwell membrane were detached by trypsin for quantification using the CellTiter-Glo assay against a standard curve for cell number. Percentage invasion or migration was determined by measuring the number of cells that had invaded or migrated to the bottom chamber, divided by total number of cells seeded.

### RNA sequencing and gene set enrichment analyses

RNA was extracted using the RNeasy Plus Mini Kit (QIAGEN) following manufacturer’s protocol. Yield and integrity of RNA were determined using the Agilent 2100 Bioanalyzer (Agilent Technologies) following manufacturer’s protocol. Sample preparation for the Bioanalyzer was done using the Agilent RNA 6000 Nano Kit (Agilent Technologies, USA) following manufacturer’s protocol. RNA sequencing was done at the Next Generation Sequencing Platform in the Genome Institute of Singapore, A*STAR. All pre- and post-processing steps, including bioinformatics analyses were performed by the sequencing platform. A list of read counts was provided as the final output of the sequencing. Differentially expressed genes were obtained using the DESeq2 package [43]. Subsequently, GSEA using hallmark gene sets from the Molecular Signatures Database (MSigDB) [44, 45] was done using the fgsea package [46]. All packages and pipelines were processed using R on RStudio.

### Single-cell RNA sequencing analyses

In-house-generated single-cell datasets from HCT116 PAR/LD/MD/HD cells were aligned to the human reference genome (GrCh38). Cellranger count was used to generate raw barcode matrices from the fastq files through the Cellranger 6.0.1 pipeline. The raw feature-barcode matrix was loaded into Seurat (v4.3.0). Cells of low quality (fewer than 200 genes per cell) and cells expressing rare genes (those expressed in fewer than 30 cells) were excluded from the analysis. Cells with over 20% mitochondrial gene content were also removed. Potential cell doublets were filtered out by first setting an upper limit of 6000 genes detected per cell and then consequently, by using the DoubletFinder package. The data was normalized with ScaleData() using a scaling factor of 10000, and gene expression was scaled to unit variance and a mean of 0. Principal component analysis was performed using the RunPCA function for dimensionality reduction, with an elbow plot determining the inflection point where variance changes became insignificant. The neighborhood graph for clustering was calculated using FindNeighbors, and cell clustering was done with the FindClusters function using Louvain clustering. UMAPs of the clusters were made using RunUMAP() function, and split UMAPs were used to compare the differences in clusters across different conditions. Proportions of cell clusters across different conditions were visualized using ggplot2. Signature scores were calculated using the UCell package. Lastly, the expression of RESIST-M signatures was verified across the different LD/MD/HD conditions using DotPlot() or VlnPlt() function.

### Quantitative RT-PCR

RNA was extracted as per previously described. Nanadrop was used to determine the concentration and quality. cDNA was synthesized using SuperScript IV VILO Master Mix (Invitrogen) following manufacturer’s protocol. qPCR was done using the KAPA SYBR FAST qPCR Master Mix (2X) Kit (KAPA BIOSYSTEMS) following manufacturer’s protocol, and qPCR cycles were done on QuantStudio 7 Flex Real-Time PCR System (Applied Biosystems). Primers were purchased from Integrated DNA Technologies, Singapore. Primer sequences are found in Table 1. All qPCR runs were done with technical quadruplicates and three independent biological replicates.

**Table 1:** Sequences for qPCR primers.

Gene	Oligo Sequence (5′ – 3′)
SERPINE1_F	GCAACGTGGTTTTCTCACCC
SERPINE1_R	CTCTAGGGGCTTCCTGAGGT
VIMENTIN_F	TCTCTGAGGCTGCCAACCG
VIMENTIN_R	CGAAGGTGACGAGCCATTTCC
ACAT2_F	GCCTTGCAGTCCAGTCAATA
ACAT2_R	CTCAAGTAAGCCAAGTGAGGAG
HMGCS1_F	TATAGCTCTAGGTGTGCTCCTG
HMGCS1_R	CCTCATCCACACCTCCAATAAC
HMGCR_F	TTGCTTGCCGAGCCTAAT
HMGCR_R	GTTTCAGTCACCAACCTCCT
MVD_F	CCCAATGCCGTGATCTTCA
MVD_R	TCAGAAACGTGTCTCCATTCG
CYP51A1_F	CTCATCGCTCTTGCCAAATAAG
CYP51A1_R	TCCATCCTCACACACACAATAA
INSIG2_F	CTGATGCACAAGGGCAAGAT
INSIG2_R	GACACATCCTCTCACTCACTCC
SMARCD3_F	GATCAGTGCTCTGGACAGTAAG
SMARCD3_R	CTCTGGAGAAGCTTAGCATGAA
SC5DL_F	TCACTTTGTGGGATAGGATTGG
SC5DL_R	CGCTTTCCCTCTGTCATCTC
BACTIN_F	CACCATTGGCAATGAGCGGTTC
BACTIN_R	AGGTCTTTGCGGATGTCCACGT
GAPDH_F	AAGGGCATCCTGGGCTACACTGAG
GAPDH_R	GAAATGAGCTTGACAAAGTGGTCGTT

### ELISA

Measurement of PAI-1 (BMS2033, Thermo) and TGF-β1 (ab100647, abcam) in cell culture supernatant was performed following manufacturer’s protocol. A suitable number of cells were first seeded in 6-well plates supplemented with complete media. 24 to 48 h later, spent media were replaced with serum-free media and incubated for a further 24 to 48 h. Supernatant was collected for downstream ELISA assays. Cells were lysed in RIPA buffer and subjected to protein quantification using BCA assay for normalization.

### Western blot

Western blot was done as per standard protocol. Briefly, cells were lysed in RIPA buffer (89901, Thermo) supplemented with Protease Cocktail Inhibitor (4693116001, Sigma) and PhosSTOP (4906837001, Sigma). Lysates were cleared by centrifugation and subjected to protein quantification using the Pierce BCA Protein Assay Kit (23225, Thermo) following manufacturer’s protocol. NuPAGE LDS sample buffer (NP0008, Invitrogen) supplemented with NuPAGE reducing agent (NP0009, Invitrogen) was added to the protein lysates and subjected to SDS-PAGE. Wet transfer was done onto PVDF membrane (IPFL85R, Merck) and blocked for 1 h with TBS blocking buffer (927-60001, LI-COR) or 1.5% non-fat milk (#1706404, Biorad), and subsequently incubated with primary antibodies overnight at 4 °C. NIR imaging was done using the LI-COR Odyssey CLx system and Image Studio Ver 4.0. Chemiluminescence imaging was done using the Azure 600 (Azure Biosystems) after incubation with HRP substrate (K-12042-D20, Advansta). List of antibodies and dilutions used is found in Table 2.

**Table 2:** List of antibodies.

Antibody	Manufacturer	Cat #	Dilution
PAI-1	RnD Systems	MAB1786	1:500
SMAD 2/3	CST	8685	1:1000
p-SMAD2 (S465)	abcam	ab216482	1:1000
p-SMAD3 (S434 + S425)	abcam	ab52903	1:1000
SMAD4	abcam	ab40759	1:1000
Pan-ERK	CST	4695	1:1000
p-ERK 1/2	Thermo	PA1-4607	1:1000
Clathrin	abcam	ab21679	1:1000
TGF-β RII	CST	79424	1:1000
GAPDH	Proteintech	10494-1-AP	1:5000
Caveolin-1	abcam	ab2910	1:1000
MEK 1/2	CST	4694	1:1000
p-MEK 1/2	CST	2338	1:1000
p-AKT	CST	4060	1:1000
Histone H3	CST	4499	1:1000
Cleaved- Caspase 3	CST	9661	1:1000
Anti-Ms 680RD	LI-COR	926-68070	1:10 000
Anti-Rb 680RD	LI-COR	926-68071	1:10 000
Anti-Ms 800CW	LI-COR	926-32210	1:10 000
Anti-Rb 800 CW	LI-COR	926-32211	1:10 000
Anti-Rb HRP	CST	7074	1:10 000
Anti-Ms HRP	CST	7076	1:10 000

### ATAC-seq sample preparation and data analyses

Sample preparation for ATAC-seq of HCT116 parental and oxaliplatin-resistant models was done using the ATAC-seq kit following manufacturer’s protocol (53150, ACTIVE MOTIF). Quality of libraries was confirmed using the Agilent DNA Bioanalyzer. Sequencing adapters were trimmed using Skewer (v0.2.2), and quality check was done using fastqc (v0.11.5). Reads were mapped to the GrCh38 reference genome using BWA (v0.7.12-r1039). A Pearson correlation between samples was calculated using deepTools (v3.0.2). Peak calling was performed using MACS2 (v2.1.2). Bigwig files were generated and used to plot ATAC-seq peaks in target genes using IGV and deeptools/pyGenomeTracks. The position frequency matrix for SMAD-binding motif (MA0513.1) was used as an input to identify occurrence of the SMAD motif in promoter regions of SERPINE1 and SMARCD3, denoted as −1kb to +1 kb from start of 5′ UTR region, using the FIMO tool in Meme suite (v5.5.7).

### In vivo modeling of therapy-induced evolution of drug-resistance and metastasis

#### Animals

Six to eight weeks old male NSG (NOD.Cg-Prkdc^scid^ IL2rg^tm1Wjl^/SzJ) immunocompromised mice purchased from Invivos Pte Ltd (Singapore) were used in all animal studies. Animals were group housed in individually ventilated cages in the Biological Resource Centre, A*STAR, Department 3. Room lighting was set to a 12-h light-dark cycle as recommended by the National Advisory Committee for Laboratory Animal Research (NACLAR). Animals were provided with irradiated Altromin 1324 diet and autoclaved water, *ad libitum*. The protocol was approved by the Biological Resource Centre Animal Use and Care Committee (IACUC #181372), and animals were maintained in accordance with guidelines from the American Association of Laboratory Animal Care (AALAC). No animals were excluded from any studies unless specified. Sex is not a biological variable for our studies.

#### Tumor growth kinetics and spontaneous metastasis

Each mouse was injected subcutaneously in the left flank with 0.5 to 1 million cells resuspended in serum-free McCoy’s media and 50% Matrigel (354234, Corning). Tumor growth was tracked weekly by measuring using vernier calipers. Tumor volume was determined by (L × W^2^)/2, where L is the longest axis and W is the perpendicular axis. Eight weeks post inoculation, resection of the primary tumor was done under anesthesia, and mice were left to recover with proper post-operative care. Lungs were then harvested to assess for metastasis three to four weeks after surgery. Lungs were first perfused with 10% neutral buffered formalin (Sigma) during harvesting and fixed in the same fixative for at least 48 h. Paraffin blocking, sectioning, and relevant H&E staining were done at the Advanced Molecular Pathology Laboratory (AMPL) in the Institute of Molecular & Cell Biology (IMCB), A*STAR. Tumor measurements were not blinded.

#### In vivo effects of oxaliplatin treatment on spontaneous metastasis

Mice were injected with treatment naïve HCT116 or SW480 cells as described previously. Clinical grade oxaliplatin was kindly provided for by Dr Clarinda Chua from the National Cancer Center of Singapore. Drug was diluted with water for injection (B.Braun) to a final concentration of 1 mg/mL before treatment. Mice were randomized when the average cohort tumor size reached 200–300 mm^3^. Treatment groups received 5 mg/kg of oxaliplatin weekly, *i.p*., for 5 consecutive weeks, while vehicle group was injected with water for injection at 0.1 ml per 10 g of body weight. Tumor resection was done 1-week after the final dose of treatment, and animals were sacrificed 3 to 4 weeks after surgery for organ harvesting as per previously described. Drug treatments and tumor measurements were not blinded.

#### Impact of concomitant administration of tiplaxtinin and oxaliplatin on metastasis

Mice were injected with naïve or parental HCT116 cells and randomized as described previously. Tiplaxtinin (CAS # 393105-53-8, Tocris or Selleckchem) was dissolved in DMSO to a concentration of 50 mg/mL, and then added to corn oil (Sigma) to a final concentration of 5 mg/mL. Treatment group received 20 mg/kg of tiplaxtinin daily (Monday to Friday), *p.o*., for 5 consecutive weeks. Vehicle groups received only corn oil added with equal amounts of DMSO at 0.1 ml per 10 g of body weight. All mice for this experiment received oxaliplatin at 5 mg/kg once a week, *i.p*., for 5 consecutive weeks. Tiplaxtinin treatment began simultaneously with the first dose of oxaliplatin. Tumor resection was done 1-week after the final dose of treatment, and animals were sacrificed three to 4 weeks after surgery for organ harvesting as per previously described. Drug treatments and tumor measurements were not blinded.

#### In vivo effects of simvastatin treatment on oxaliplatin-resistant tumor models

Mice were injected subcutaneously with either parental HCT116 (HCT116-PAR) or the oxaliplatin-resistant cell line models (HCT116-LD, MD, HD). Once the tumor size reached ~200 mm^3^ to 300 mm^3^, mice were randomized into either control or drug treatment groups for each cell line as described previously. Control and drug treatment groups received DMSO and simvastatin, respectively. Simvastatin (CAS # 79902-63-9, Tocris) was dissolved in DMSO to a concentration of 50 mg/mL, and then added to corn oil (Sigma) to a final concentration of 2 mg/mL. Drug treatment group received 10 mg/kg of simvastatin daily by oral gavage. Vehicle group received equal amounts of DMSO in corn oil. Drug treatments and tumor measurements were not blinded.

### Slide scan acquisitions and image analyses

Tissue slides were scanned using the Vectra Polaris Automated Quantitative Pathology Imaging System (Akoya Bioscience) and analyzed using both inForm Tissue Finder version 2.4 and Phenochart version 1.0 software by Akoya Bioscience. Slides were scanned at 20× magnification and exported as TIFFs to be analyzed with InForm.

To determine the severity of pulmonary metastasis, image analysis of tumor nodules and normal lung tissue areas was quantified using the threshold algorithm in InForm. Metastasis score is calculated using the following formula: (Area of all nodules/Total area of tissue) × 100.

### shRNA genetic knockdown

To generate stable knockdown lines, suitable bacteria clones from the RNAi Consortium (TRC) shRNA Library (Broad Institute) were selected and sub-cultured in terrific Broth (CUS-4051-1L, Axil Scientific) supplemented with 1X carbenicillin (ThermoFisher). Plasmids were extracted using the Plasmid Plus Midi kit (QIAGEN) or Plasmid Plus Maxi kit (QIGEN) following manufacturer’s protocol. Purity was determined using the Nanodrop 8000.

Custom shRNAs against PAI-1 were designed using the BLOCK-iT™ RNAi Designer (ThermoFisher) and cloned into the pLKO.1 vector. The TRC cloning vector was a gift from David Root (Addgene plasmid #10878; RRID: Addgene_10878). Lentivirus was generated in HEK293T cells using Lipofectamine 3000 Transfection kit (Thermo) according to manufacturer’s protocol. A total of 9 μg of shRNA plasmid and 3 μg each of the third-generation lentiviral packaging plasmids were used for the transfection mix. The three lentiviral plasmids, pMD2.G (Addgene plasmid #12259; RRID: Addgene_12259), pMDLg/pRRE (Addgene plasmid #12251; RRID: Addgene_12251), and pRSV-Rev (Addgene plasmid #12253; RRID: Addgene_122-53) were gifts from Didier Trono. Transfection lasted for about 16 to 24 h before a change to appropriate, fresh media. After 24 h, supernatant containing the viral particles was collected and centrifuged at 1000 rpm for 5 min and filtered using a 0.45 μm filter before adding onto target cells supplemented with 1X polybrene (Santa Cruz). Cells were transduced for 24 to 48 h. Puromycin selection was then done for at least two passages to establish stably transduced cell lines. Knockdown of target genes was validated with qPCR, western blot, and/or ELISA. Sequences of control and PAI-1 shRNAs used are listed in Table 3.

**Table 3:** Sequence of shRNA used for knockdown.

Name	Oligo Sequence (5′– 3′)	Notes
shControl_F	CCGGTCTCGCTTGGGCGAGAGTAAGCTCGAGCTTACTCTCGCCCAAGCGAGATTTTTG	
shControl_R	AATTCAAAAATCTCGCTTGGGCGAGAGTAAGCTCGAGCTTACTCTCGCCCAAGCGAGA	
PAI1 sh3	CCGGCAGACAGTTTCAGGCTGACTTCTCGAGAAGTCAGCCTGAAACTGTCTGTTTTTG	TRCN0000052271, NM_000602.1-1041s1c1
PAI1 sh5_F	CCGGGCTGACTTCACGAGTCTTTCACTCGAGTGAAAGACTCGTGAAGTCAGCTTTTTG	
PAI1 sh5_R	AATTCAAAAAGCTGACTTCACGAGTCTTTCACTCGAGTGAAAGACTCGTGAAGTCAGC	

### Immunofluorescence staining

Cells were seeded and cultured in standard cell culture conditions for 24 h prior to staining on 8-well imaging slides (Ibidi). Lipid raft staining was done using the Vybrant Alexa Fluor 555 lipid raft staining kit (ThermoFisher) according to manufacturer’s protocol. Cells were then subsequently co-stained with primary antibody using TGFbRII (CST) and secondary antibody using AF647 (Thermo). Images were taken using the Nikon Ti-E TIRF System.

### Lipid rafts sucrose density fractionation

Lipid raft fractionation protocol was adapted from Zuo et al. [47]. Briefly, cells were seeded and grown to near confluence in four 100-mm dishes. During harvesting, cells were washed twice with ice-cold PBS and scraped with 0.75 mL of 500 mM sodium carbonate, pH 11.0, and incubated on ice for 10 min. Mechanical homogenization was done using 10 strokes of a Dounce homogenizer followed by three 20-s bursts of sonication on ice. Homogenates were adjusted to 42.5% sucrose by adding equal volumes of 85% sucrose in 2X HBS (25 mM HEPES, pH6.5, 150 mM NaCl) in an ultracentrifuge tube (344060, Beckman Coulter). 3.5 mL of 35% and 5% sucrose were overlayed to create a discontinuous sucrose gradient. Samples were subjected to ultracentrifugation at 260,000 × *g* for 16 h at 4 °C using the Optima XPN-80 and SW40Ti rotor (Beckman Coulter). Twelve 1-mL fractions were collected from the top and subjected to immunoblotting.

### Patient cohorts and survival analyses

#### Datasets

Normalized bulk RNA-sequencing data for TCGA-COADREAD, TCGA-COAD and -READ datasets were obtained from UCSC Xena browser. Normalized counts for bulk RNA-sequencing data were obtained for PETACC-3 dataset as described [30] and SG-BULK dataset (Synapse ID: syn26720761). Robust Multichip Average/Frozen robust multiarray analysis (RMA/FRMA) normalized microarray datasets [28] (GSE14333, GSE37892, GSE39582) were obtained from Synapse ID: syn2634742.

#### Analyses of transcriptomic data from public datasets

Boxplots for gene signatures across CMS subtypes are shown for TCGA-COADREAD data (*n* = 377, with CMS annotations) and PETACC-3 data (*n* = 604). CMS annotations for patients in TCGA-COADREAD data were extracted from Colorectal Cancer Subtyping Consortium (CRCSC) available at Synapse ID: syn2623706. The CMS was calculated with the reference CMS classifier (https://github.com/Sage-Bionetworks/CMSclassifier) for PETACC-3 dataset. Gene signatures shown include RESIST-M1(*SERPINE1, SMARCD3*), RESIST-M2 (*SC5D, FDPS, MVD, HMGCS1, HMGCR, CYP51A1, ACAT2*), Yin et al. [31] (*P4HA1, ATF6, PHLDB3, IBTK, COPE*), Lin et al. [32] (*ALCAM, CD22, CASP1, CISH*).

Heatmap comparing RESIST-M genes to reported gene signatures of oxaliplatin resistance (Yin et al., Lin et al.), CRC recurrence [33] (RCC), and DNA repair gene signature [34] (RPS) was done using publicly deposited bulk RNA-seq data from Singapore colorectal cancer patients (*n* = 162, SG-BULK, Project SynID: syn26720761 [29]). Genes were tabulated using the available clinical annotations for iCMS, CMS, TGFBR2 mutation, iCMS-microsatellite (iM), or iCMS-fibrosis status (iF). Salmon TPM counts of RESIST-M signature genes (*SERPINE1, SMARCD3*, *SC5D, FDPS, MVD, HMGCS1, HMGCR, CYP51A1, ACAT2*) and rest of the signatures were z-scored using scale() function in R, and heatmap was made using heatmap() function using R version 4.2.2. Patients with high expression of both *SERPINE1* and *SMARCD3*, along with low expression of *SC5D, FDPS, MVD, HMGCS1, HMGCR, CYP51A1*, and *ACAT2* correlated predominantly with an CMS4-iCMS3-MSS-fibrotic subtype. RCC gene score includes expression of twelve genes, out of which seven are used for analyses in this paper. The seven genes include stromal genes (*INHBA, BGN, FAP*), cell cycle genes (*MKI67, MYC, MYBL2*) and *GADD45B* [33]. RPS gene score includes expression of four DNA repair genes- *RIF1, XRCC5, PARPBP, RAD51* [34].

Kaplan-Meier (KM) survival curves were generated to evaluate the prognostic significance of gene signatures: RESIST-M1, RESIST-M2, Yin et al., Lin et al., RCC, and RPS using TCGA-COADREAD dataset. Overall survival (OS) of patients was stratified into high- and low-expression groups based on median expression levels. Survival analysis was performed using the log-rank test, and hazard ratios were calculated using the Cox proportional hazards model.

KM curves plotted for RESIST-M1 and -M2 signatures together are represented by median of all signature genes expression (9 genes: *SC5D, FDPS, MVD, HMGCS1, HMGCR, CYP51A1, ACAT2, SERPINE1, SMARCD3*) in the sample. For genes *SMARCD3* and *SERPINE1*, a reciprocal is done with the following formula, before combining with other genes to get the median expression:

min + (max − min) * (max − expression)/(max − min). Each sample will be assigned a high [1] or low (0) status depending on if its expression is higher or lower than the median signature gene expression threshold. Median signature gene expression threshold is obtained from median of all samples' signature expression. Kaplan–Meier survival plot is using R “ggsurvplot” and “survfit” function with log-log confidence interval type.

Gene expression data were obtained from the PETACC-3 trial as previously described [30]. The M1 and M2 scores were obtained by calculating the mean expression of M1 and M2 genes for each patient. The score was considered “high” if above the median, “low” otherwise. Survival was modeled with Cox regression and the log-rank test. We used the survminer package to plot Kaplan-Meier curves. The CMS was calculated with the reference CMS classifier (https://github.com/Sage-Bionetworks/CMSclassifier) for PETACC-3 dataset. *P*-values were considered significant if below 0.05.

### Synthetic lethal drug screen

About 3000 cells were seeded in each well of black 384-well plates and incubated under standard cell culture conditions for 48 h. Culture media was then refreshed in the morning, and drugs from the Anti-Cancer (L3000, Selleck Chemicals, USA) and Kinase Inhibitor (L1100, Selleck Chemicals, ISA) compound library were added at a concentration of 1 µM to each well in the afternoon. Cells were treated for 72 h, and cell viability was determined using CellTiter-Glo (Promega) following manufacturer’s protocol. Data obtained was analyzed using EARO High-throughput Phenomics Database Portal. Percentage inhibition was calculated luminescence value of drug treated wells over DMSO control.

### Statistical analyses

All experiments were performed in at least three biological replicates, and data for descriptive statistics are expressed as standard error of mean (s.e.m.) unless otherwise stated. Methods of statistical analysis are described in text. A *p* value of <0.05 is considered statistically significant. All analyses were done with GraphPad PRISM v9 and v10.

## Supplementary information

Supplementary Figure S1

Supplementary Figure S2

Supplementary Figure S3

Supplementary Figure S4

Supplementary Figure S5

Supplementary Figure S6

Original western blots

## Data Availability

All sequencing data are deposited into the Gene Expression Omnibus database (Single Cell RNAseq: GSE299427; Bulk RNAseq: GSE299428; ATAC-seq: GSE298524). Details of other datasets used can be found in text.
