# Supplementary material for: Modeling oxaliplatin resistance in colorectal cancer reveals a SERPINE1-based gene signature (RESIST-M) and therapeutic strategies for pro-metastatic CMS4 subtype
Source: Cell Death Dis. 2025 Jul 16;16(1):529. doi: 10.1038/s41419-025-07855-y (PMC12264272; doi:10.1038/s41419-025-07855-y)
Supplement: Supplementary file 1 — Supplementary Figure S1 [file 41419_2025_7855_MOESM1_ESM.pptx]

## Slide 1
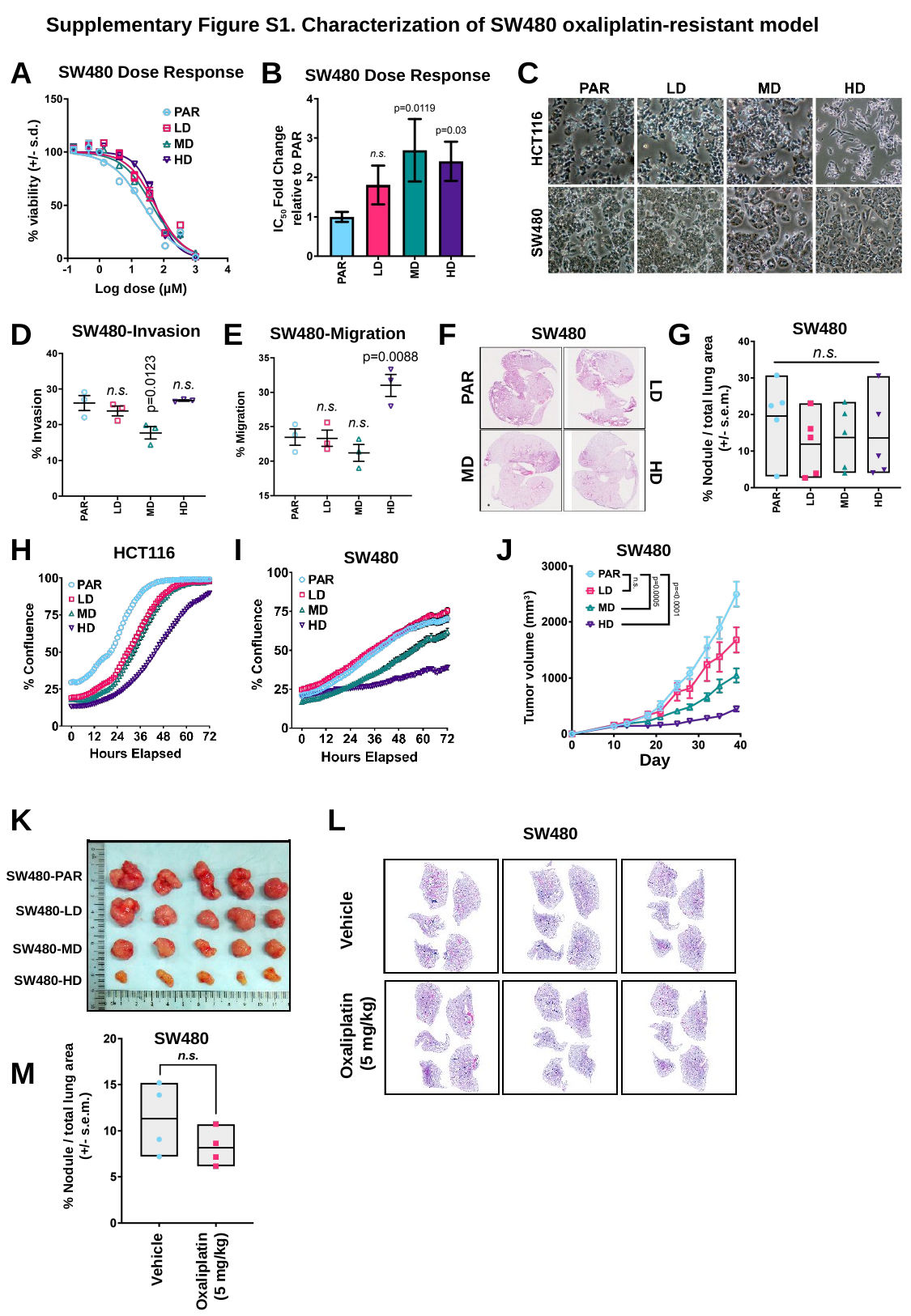

Supplementary Figure S1. Characterization of SW480 oxaliplatin-resistant model
A
B
C
SW480 Dose Response
SW480 Dose Response
IC50 Fold Change relative to PAR
% viability (+/- s.d.)
Log dose (µM)
D
E
F
G
SW480
SW480-Invasion
SW480-Migration
SW480
PAR
LD
% Nodule / total lung area (+/- s.e.m.)
% Invasion
% Migration
MD
HD
H
I
J
SW480
HCT116
SW480
Tumor volume (mm3)
Day
K
L
SW480
SW480-PAR
SW480-LD
SW480-MD
SW480-HD
Vehicle
Oxaliplatin
(5 mg/kg)
SW480
M
% Nodule / total lung area (+/- s.e.m.)
Vehicle
Oxaliplatin
(5 mg/kg)

## Slide 2
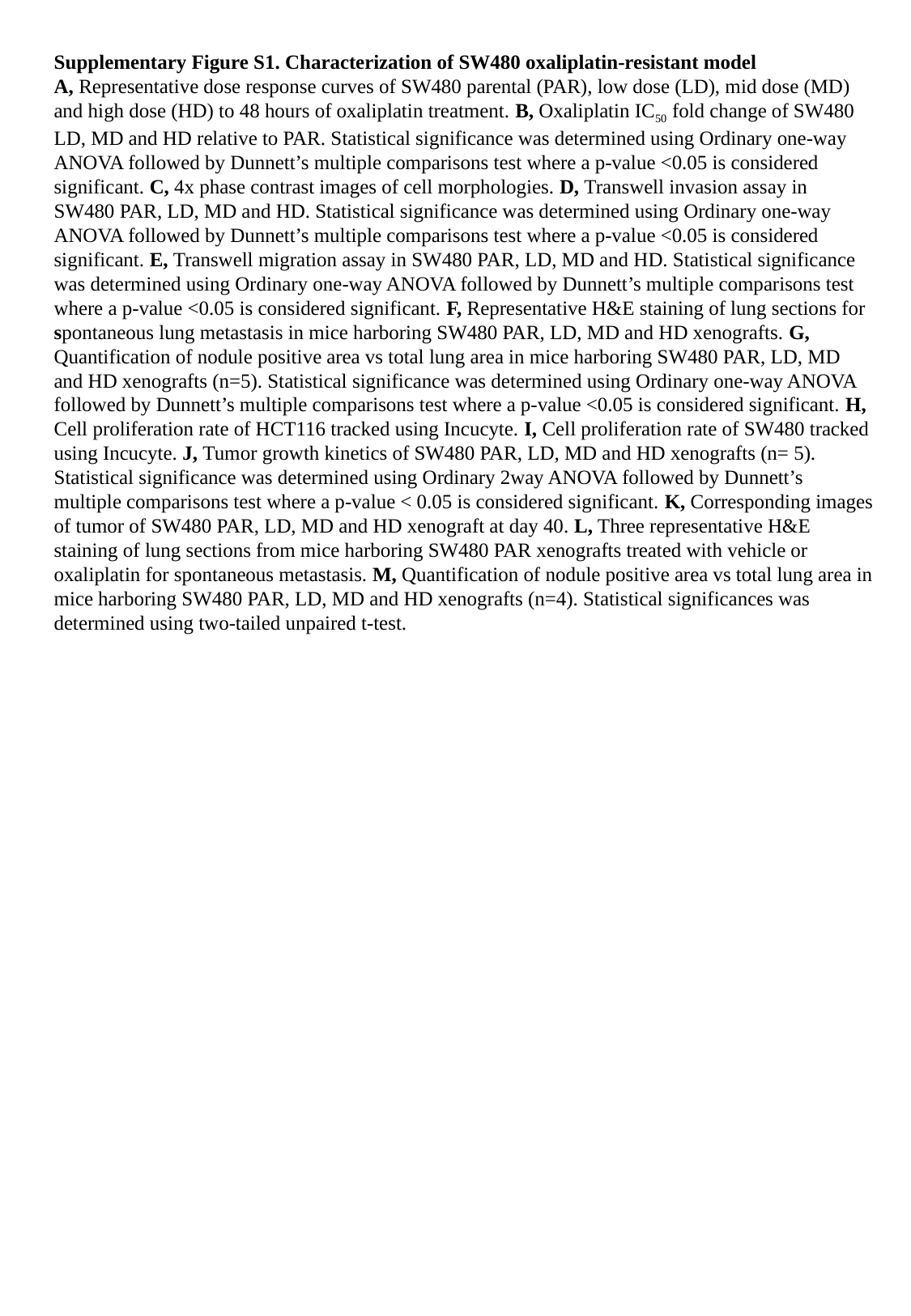

Supplementary Figure S1. Characterization of SW480 oxaliplatin-resistant model
A, Representative dose response curves of SW480 parental (PAR), low dose (LD), mid dose (MD) and high dose (HD) to 48 hours of oxaliplatin treatment. B, Oxaliplatin IC50 fold change of SW480 LD, MD and HD relative to PAR. Statistical significance was determined using Ordinary one-way ANOVA followed by Dunnett’s multiple comparisons test where a p-value <0.05 is considered significant. C, 4x phase contrast images of cell morphologies. D, Transwell invasion assay in SW480 PAR, LD, MD and HD. Statistical significance was determined using Ordinary one-way ANOVA followed by Dunnett’s multiple comparisons test where a p-value <0.05 is considered significant. E, Transwell migration assay in SW480 PAR, LD, MD and HD. Statistical significance was determined using Ordinary one-way ANOVA followed by Dunnett’s multiple comparisons test where a p-value <0.05 is considered significant. F, Representative H&E staining of lung sections for spontaneous lung metastasis in mice harboring SW480 PAR, LD, MD and HD xenografts. G, Quantification of nodule positive area vs total lung area in mice harboring SW480 PAR, LD, MD and HD xenografts (n=5). Statistical significance was determined using Ordinary one-way ANOVA followed by Dunnett’s multiple comparisons test where a p-value <0.05 is considered significant. H, Cell proliferation rate of HCT116 tracked using Incucyte. I, Cell proliferation rate of SW480 tracked using Incucyte. J, Tumor growth kinetics of SW480 PAR, LD, MD and HD xenografts (n= 5). Statistical significance was determined using Ordinary 2way ANOVA followed by Dunnett’s multiple comparisons test where a p-value < 0.05 is considered significant. K, Corresponding images of tumor of SW480 PAR, LD, MD and HD xenograft at day 40. L, Three representative H&E staining of lung sections from mice harboring SW480 PAR xenografts treated with vehicle or oxaliplatin for spontaneous metastasis. M, Quantification of nodule positive area vs total lung area in mice harboring SW480 PAR, LD, MD and HD xenografts (n=4). Statistical significances was determined using two-tailed unpaired t-test.
