# Supplementary material for: Modeling oxaliplatin resistance in colorectal cancer reveals a SERPINE1-based gene signature (RESIST-M) and therapeutic strategies for pro-metastatic CMS4 subtype
Source: Cell Death Dis. 2025 Jul 16;16(1):529. doi: 10.1038/s41419-025-07855-y (PMC12264272; doi:10.1038/s41419-025-07855-y)
Supplement: Supplementary file 2 — Supplementary Figure S2 [file 41419_2025_7855_MOESM2_ESM.pptx]

## Slide 1
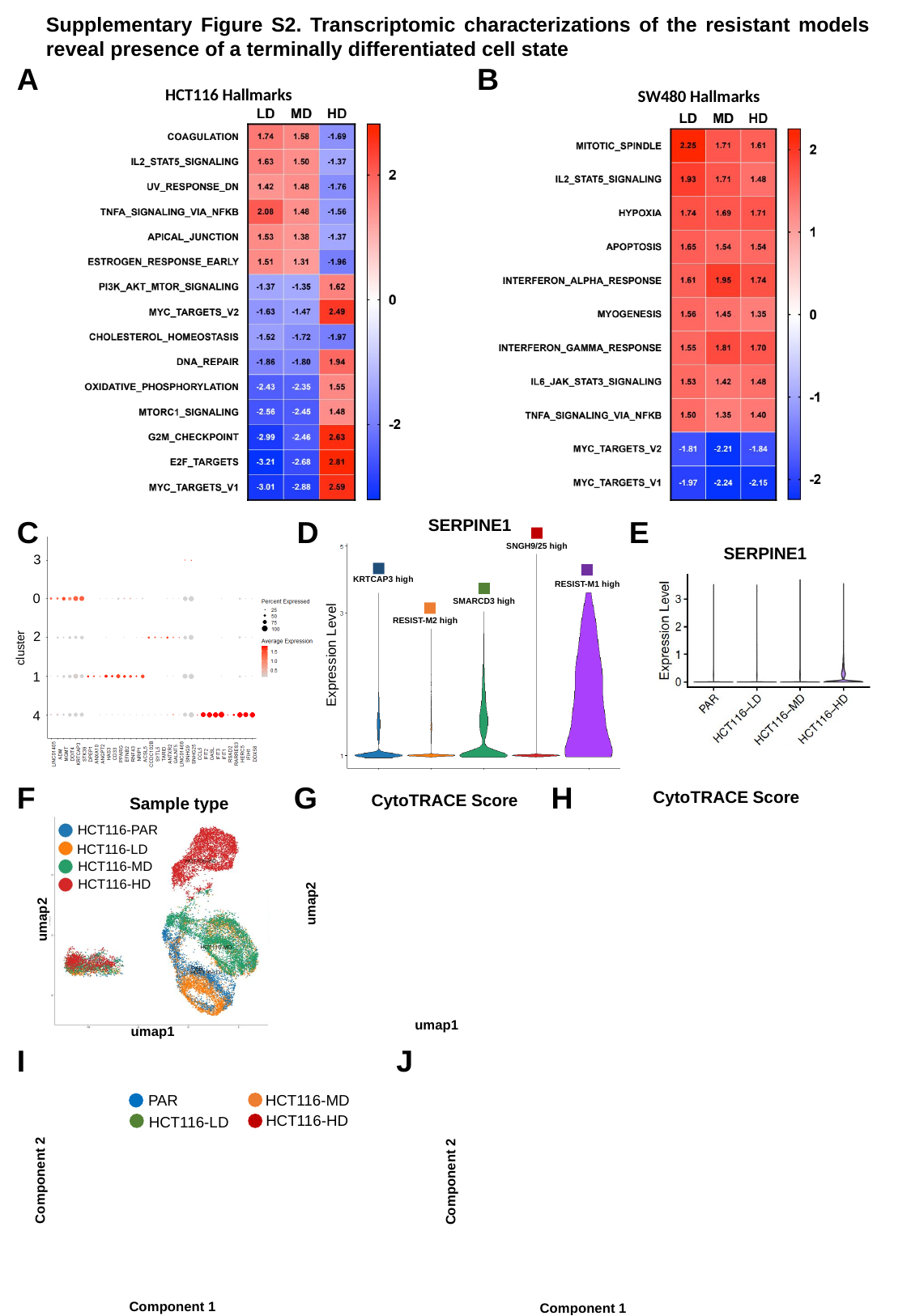

Supplementary Figure S2. Transcriptomic characterizations of the resistant models reveal presence of a terminally differentiated cell state
A
B
HCT116 Hallmarks
SW480 Hallmarks
C
D
E
SERPINE1
SNGH9/25 high
SERPINE1
3
KRTCAP3 high
RESIST-M1 high
0
SMARCD3 high
RESIST-M2 high
2
cluster
Expression Level
1
4
F
G
H
CytoTRACE Score
CytoTRACE Score
Sample type
HCT116-PAR
HCT116-LD
HCT116-MD
HCT116-HD
umap2
umap2
umap1
umap1
I
J
PAR
HCT116-MD
HCT116-HD
HCT116-LD
Component 2
Component 2
Component 1
Component 1

## Slide 2
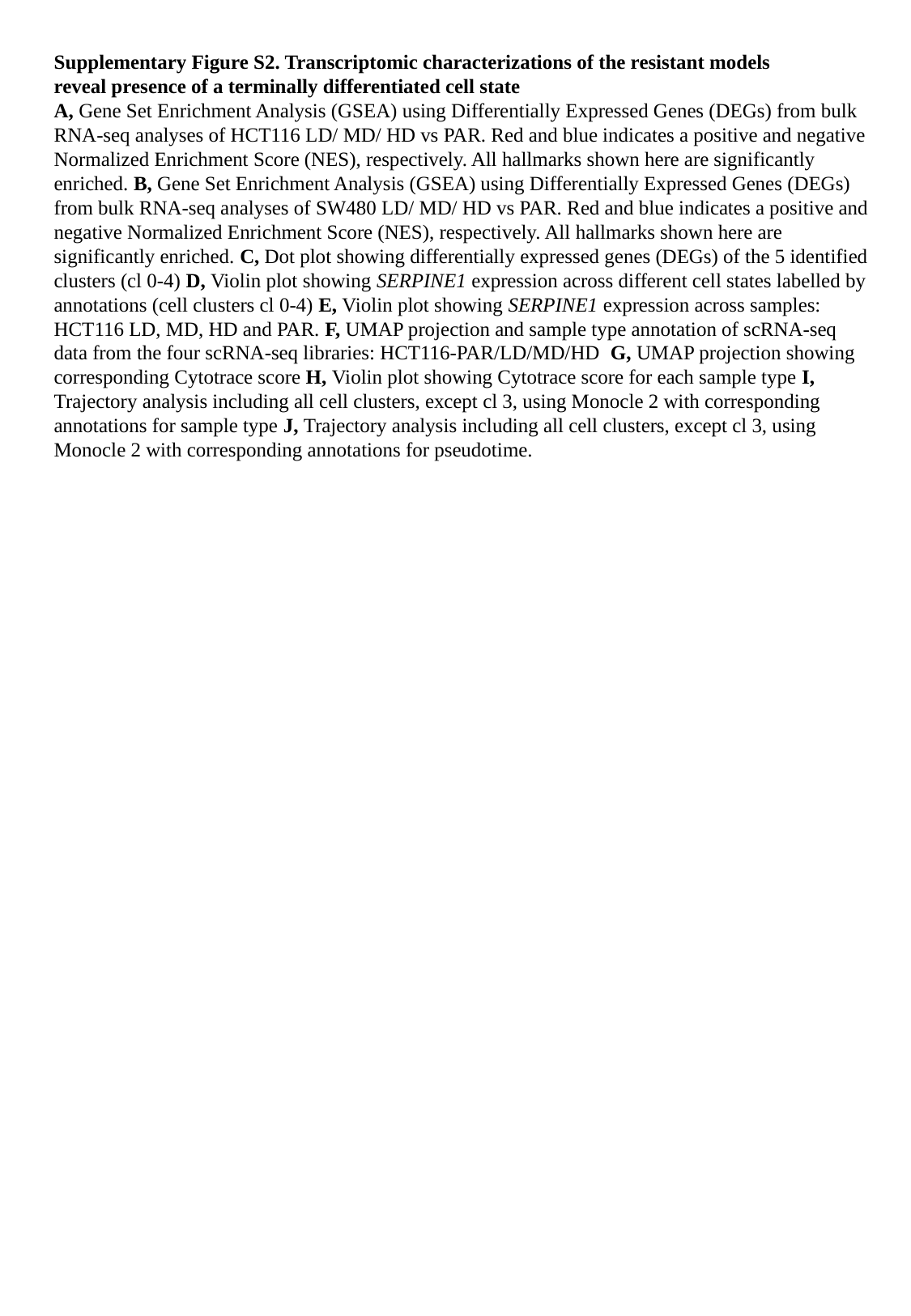

Supplementary Figure S2. Transcriptomic characterizations of the resistant models
reveal presence of a terminally differentiated cell state
A, Gene Set Enrichment Analysis (GSEA) using Differentially Expressed Genes (DEGs) from bulk RNA-seq analyses of HCT116 LD/ MD/ HD vs PAR. Red and blue indicates a positive and negative Normalized Enrichment Score (NES), respectively. All hallmarks shown here are significantly enriched. B, Gene Set Enrichment Analysis (GSEA) using Differentially Expressed Genes (DEGs) from bulk RNA-seq analyses of SW480 LD/ MD/ HD vs PAR. Red and blue indicates a positive and negative Normalized Enrichment Score (NES), respectively. All hallmarks shown here are significantly enriched. C, Dot plot showing differentially expressed genes (DEGs) of the 5 identified clusters (cl 0-4) D, Violin plot showing SERPINE1 expression across different cell states labelled by annotations (cell clusters cl 0-4) E, Violin plot showing SERPINE1 expression across samples: HCT116 LD, MD, HD and PAR. F, UMAP projection and sample type annotation of scRNA-seq data from the four scRNA-seq libraries: HCT116-PAR/LD/MD/HD G, UMAP projection showing corresponding Cytotrace score H, Violin plot showing Cytotrace score for each sample type I, Trajectory analysis including all cell clusters, except cl 3, using Monocle 2 with corresponding annotations for sample type J, Trajectory analysis including all cell clusters, except cl 3, using Monocle 2 with corresponding annotations for pseudotime.
