# Supplementary material for: Modeling oxaliplatin resistance in colorectal cancer reveals a SERPINE1-based gene signature (RESIST-M) and therapeutic strategies for pro-metastatic CMS4 subtype
Source: Cell Death Dis. 2025 Jul 16;16(1):529. doi: 10.1038/s41419-025-07855-y (PMC12264272; doi:10.1038/s41419-025-07855-y)
Supplement: Supplementary file 3 — Supplementary Figure S3 [file 41419_2025_7855_MOESM3_ESM.pptx]

## Slide 1
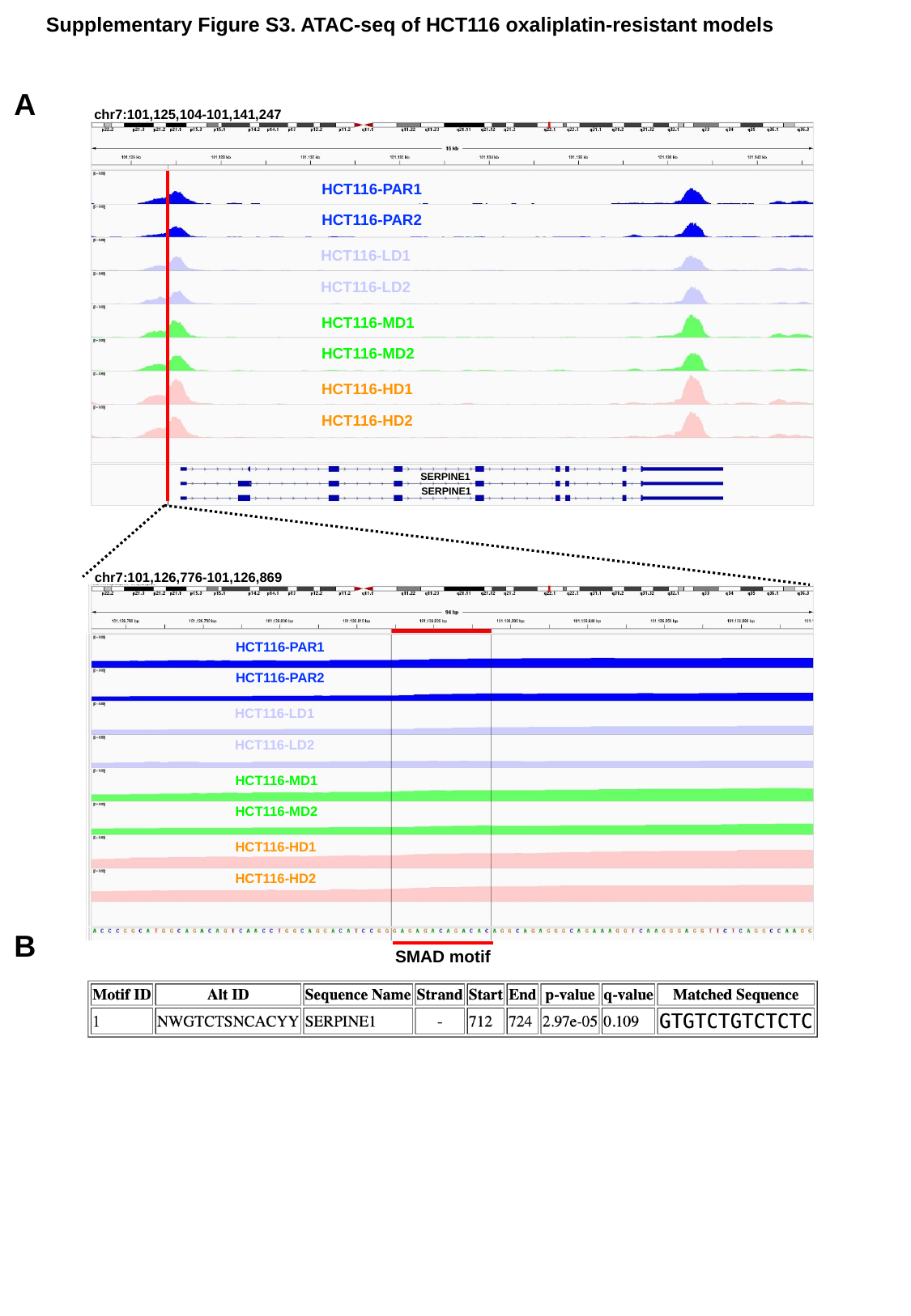

Supplementary Figure S3. ATAC-seq of HCT116 oxaliplatin-resistant models
A
chr7:101,125,104-101,141,247
HCT116-PAR1
HCT116-PAR2
HCT116-LD1
HCT116-LD2
HCT116-MD1
HCT116-MD2
HCT116-HD1
HCT116-HD2
SERPINE1
≈
SERPINE1
≈
chr7:101,126,776-101,126,869
HCT116-PAR1
HCT116-PAR2
HCT116-LD1
HCT116-LD2
HCT116-MD1
HCT116-MD2
HCT116-HD1
HCT116-HD2
B
SMAD motif

## Slide 2
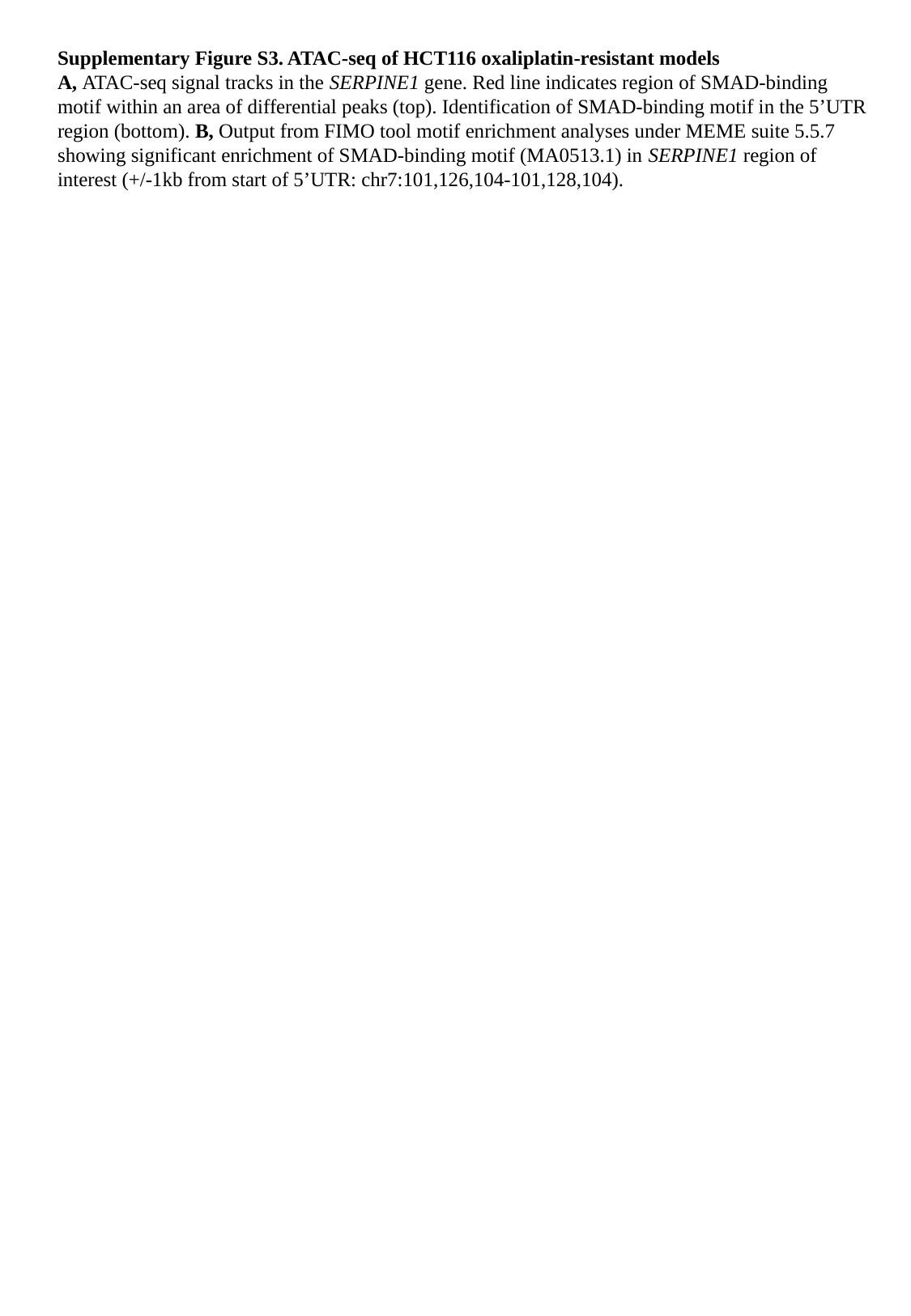

Supplementary Figure S3. ATAC-seq of HCT116 oxaliplatin-resistant models
A, ATAC-seq signal tracks in the SERPINE1 gene. Red line indicates region of SMAD-binding motif within an area of differential peaks (top). Identification of SMAD-binding motif in the 5’UTR region (bottom). B, Output from FIMO tool motif enrichment analyses under MEME suite 5.5.7 showing significant enrichment of SMAD-binding motif (MA0513.1) in SERPINE1 region of interest (+/-1kb from start of 5’UTR: chr7:101,126,104-101,128,104).
