# Supplementary material for: Modeling oxaliplatin resistance in colorectal cancer reveals a SERPINE1-based gene signature (RESIST-M) and therapeutic strategies for pro-metastatic CMS4 subtype
Source: Cell Death Dis. 2025 Jul 16;16(1):529. doi: 10.1038/s41419-025-07855-y (PMC12264272; doi:10.1038/s41419-025-07855-y)
Supplement: Supplementary file 4 — Supplementary Figure S4 [file 41419_2025_7855_MOESM4_ESM.pptx]

## Slide 1
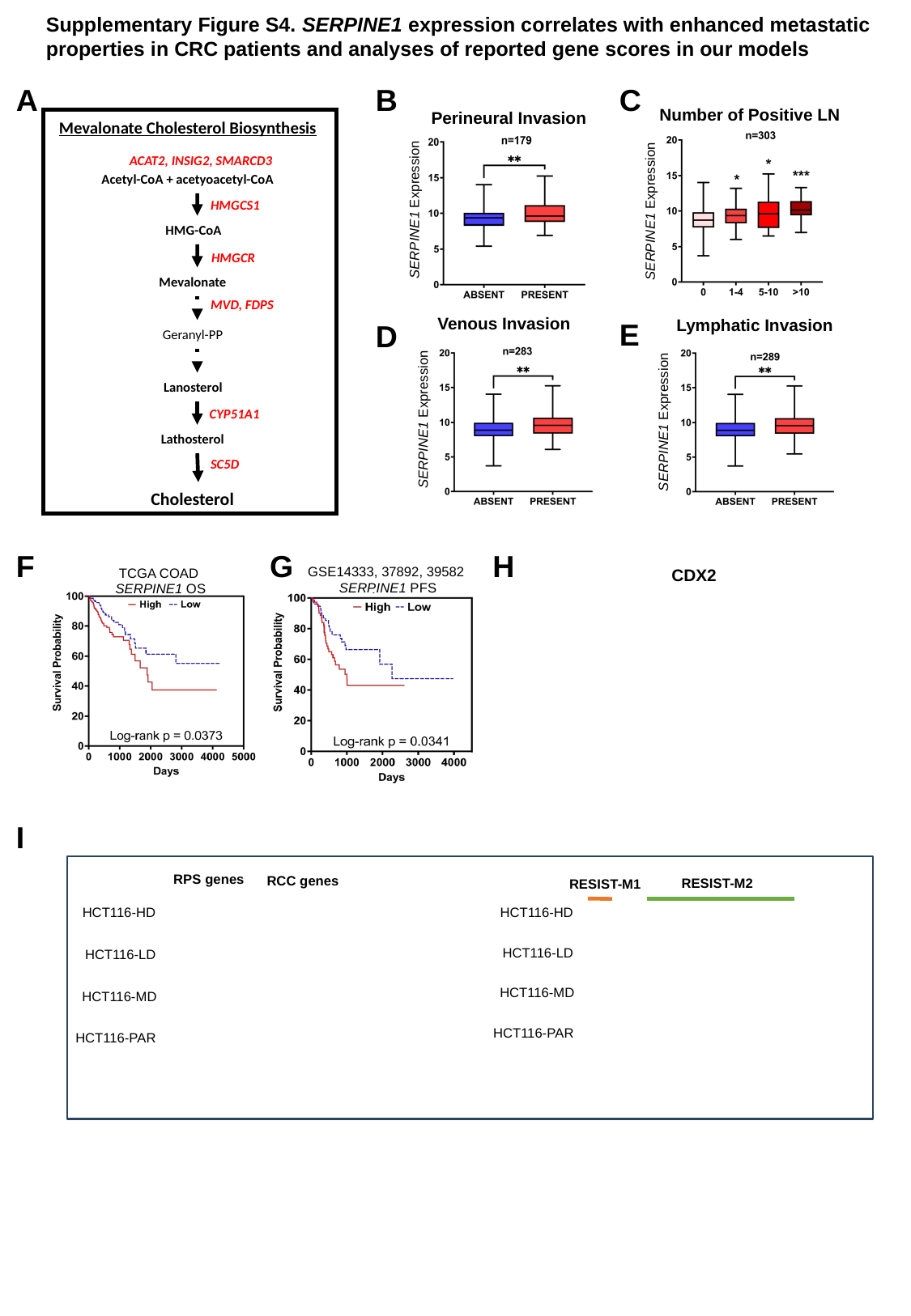

Supplementary Figure S4. SERPINE1 expression correlates with enhanced metastatic properties in CRC patients and analyses of reported gene scores in our models
A
B
C
Number of Positive LN
Perineural Invasion
Mevalonate Cholesterol Biosynthesis
ACAT2, INSIG2, SMARCD3
Acetyl-CoA + acetyoacetyl-CoA
HMGCS1
HMG-CoA
HMGCR
Mevalonate
MVD, FDPS
Geranyl-PP
Lanosterol
CYP51A1
Lathosterol
SC5D
Cholesterol
SERPINE1 Expression
SERPINE1 Expression
Venous Invasion
Lymphatic Invasion
E
D
SERPINE1 Expression
SERPINE1 Expression
F
G
H
GSE14333, 37892, 39582
SERPINE1 PFS
TCGA COAD
SERPINE1 OS
CDX2
I
RPS genes
RCC genes
RESIST-M2
RESIST-M1
HCT116-HD
HCT116-HD
HCT116-LD
HCT116-LD
HCT116-MD
HCT116-MD
HCT116-PAR
HCT116-PAR

## Slide 2
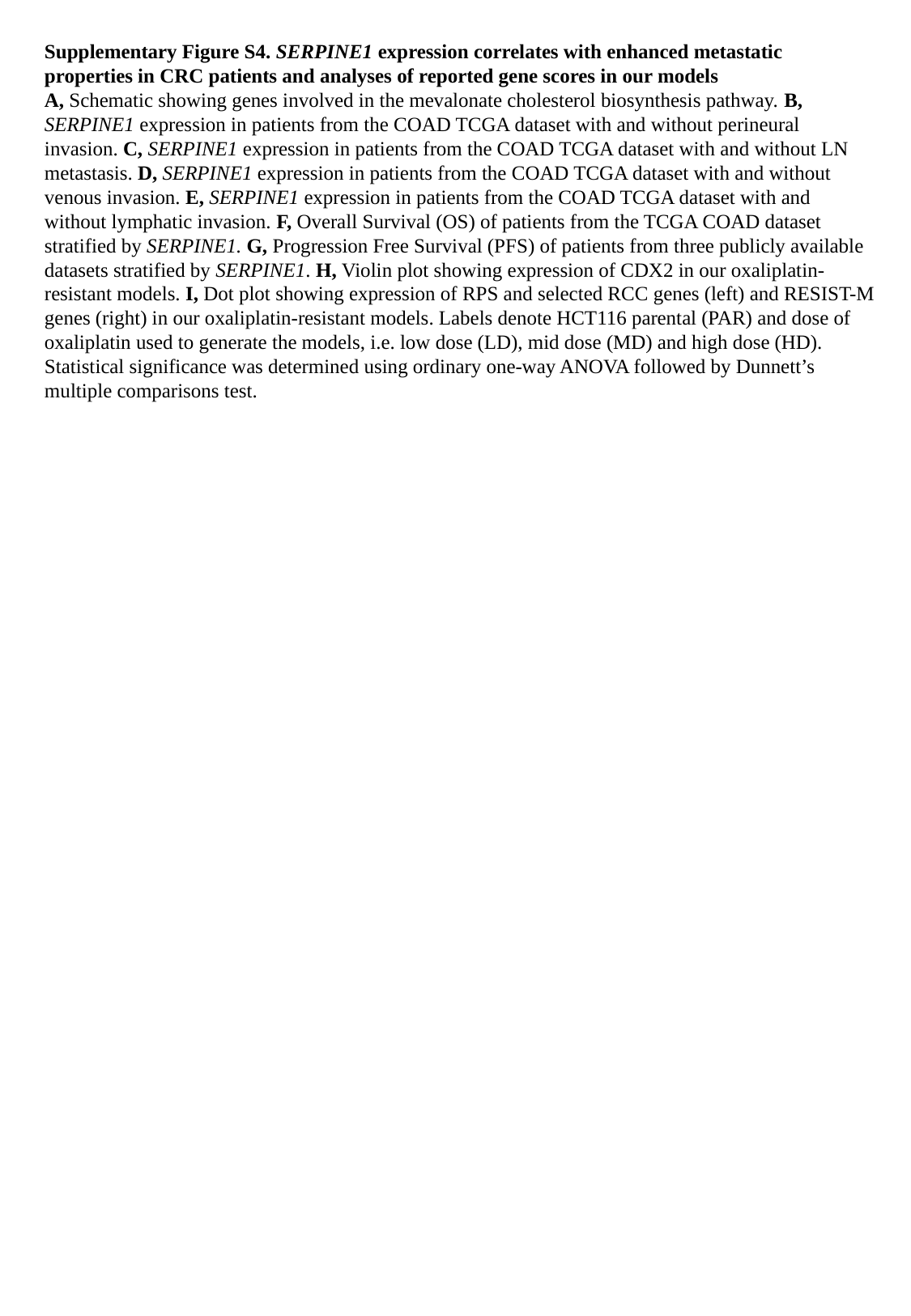

Supplementary Figure S4. SERPINE1 expression correlates with enhanced metastatic properties in CRC patients and analyses of reported gene scores in our models
A, Schematic showing genes involved in the mevalonate cholesterol biosynthesis pathway. B, SERPINE1 expression in patients from the COAD TCGA dataset with and without perineural invasion. C, SERPINE1 expression in patients from the COAD TCGA dataset with and without LN metastasis. D, SERPINE1 expression in patients from the COAD TCGA dataset with and without venous invasion. E, SERPINE1 expression in patients from the COAD TCGA dataset with and without lymphatic invasion. F, Overall Survival (OS) of patients from the TCGA COAD dataset stratified by SERPINE1. G, Progression Free Survival (PFS) of patients from three publicly available datasets stratified by SERPINE1. H, Violin plot showing expression of CDX2 in our oxaliplatin-resistant models. I, Dot plot showing expression of RPS and selected RCC genes (left) and RESIST-M genes (right) in our oxaliplatin-resistant models. Labels denote HCT116 parental (PAR) and dose of oxaliplatin used to generate the models, i.e. low dose (LD), mid dose (MD) and high dose (HD). Statistical significance was determined using ordinary one-way ANOVA followed by Dunnett’s multiple comparisons test.
