# Supplementary material for: Modeling oxaliplatin resistance in colorectal cancer reveals a SERPINE1-based gene signature (RESIST-M) and therapeutic strategies for pro-metastatic CMS4 subtype
Source: Cell Death Dis. 2025 Jul 16;16(1):529. doi: 10.1038/s41419-025-07855-y (PMC12264272; doi:10.1038/s41419-025-07855-y)
Supplement: Supplementary file 5 — Supplementary Figure S5 [file 41419_2025_7855_MOESM5_ESM.pptx]

## Slide 1
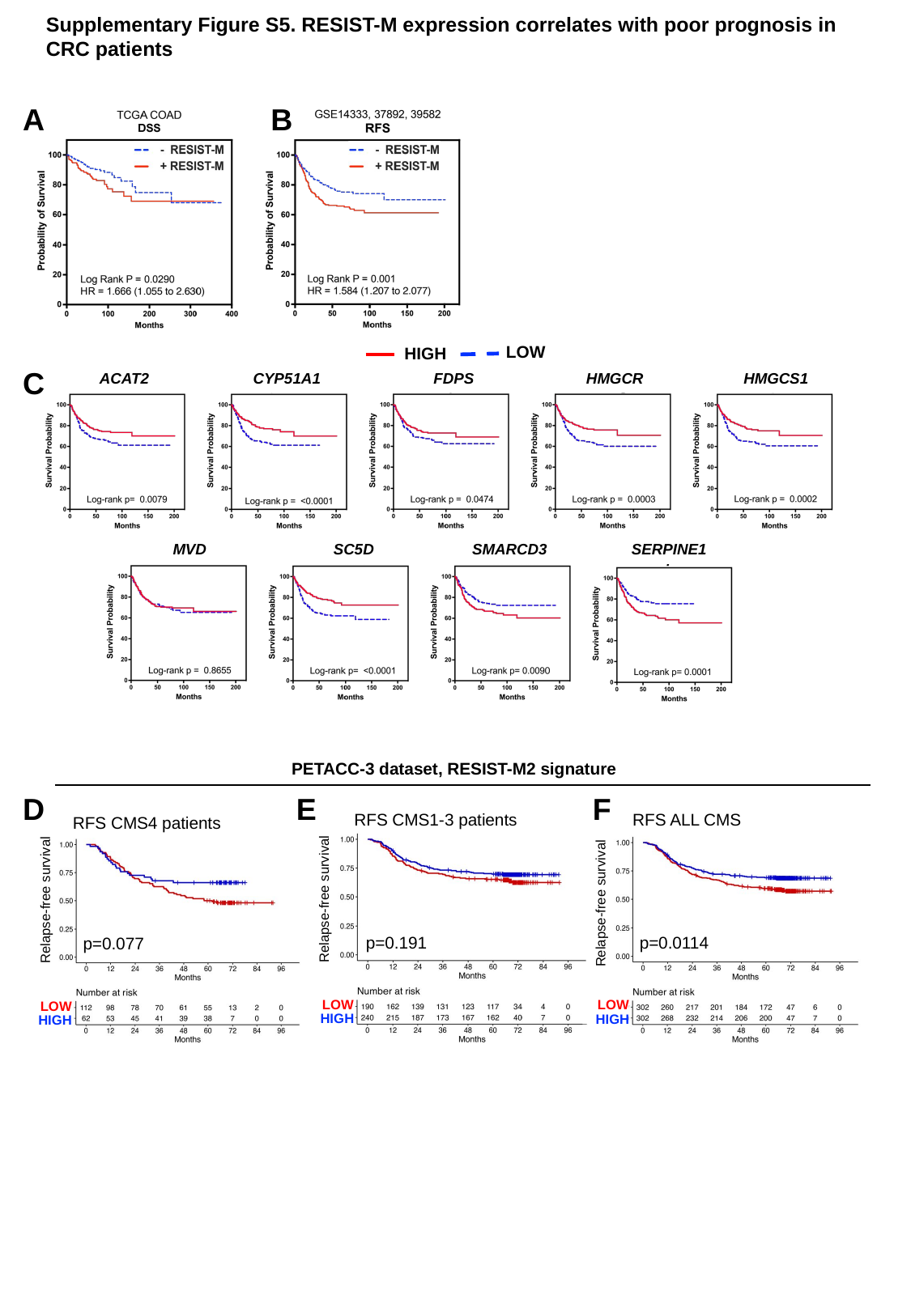

Supplementary Figure S5. RESIST-M expression correlates with poor prognosis in CRC patients
A
B
LOW
HIGH
C
ACAT2
CYP51A1
FDPS
HMGCR
HMGCS1
MVD
SC5D
SMARCD3
SERPINE1
PETACC-3 dataset, RESIST-M2 signature
D
E
F
RFS CMS1-3 patients
RFS ALL CMS
RFS CMS4 patients
Relapse-free survival
Relapse-free survival
Relapse-free survival
p=0.191
p=0.0114
p=0.077
LOW
LOW
LOW
HIGH
HIGH
HIGH

## Slide 2
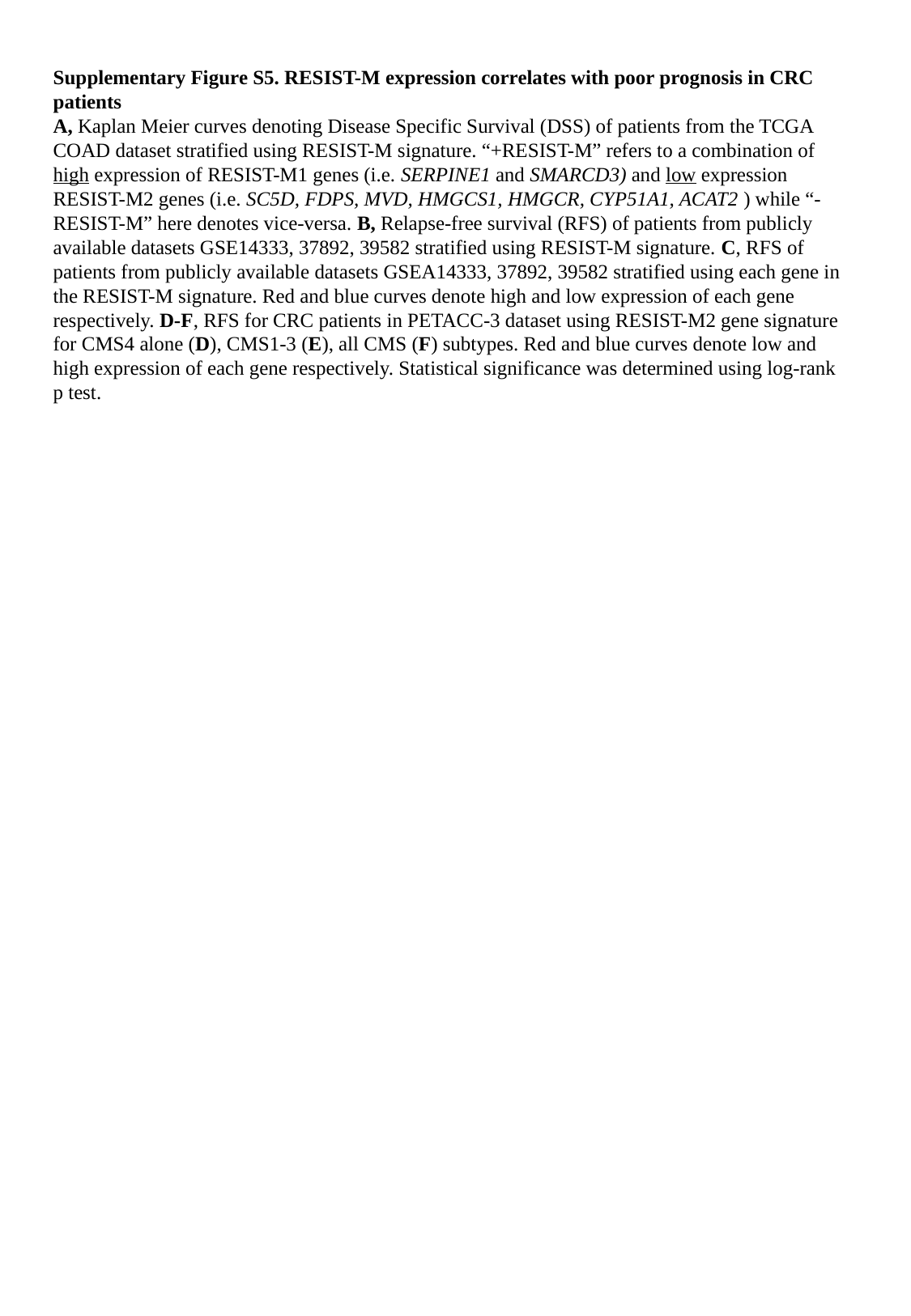

Supplementary Figure S5. RESIST-M expression correlates with poor prognosis in CRC patients
A, Kaplan Meier curves denoting Disease Specific Survival (DSS) of patients from the TCGA COAD dataset stratified using RESIST-M signature. “+RESIST-M” refers to a combination of high expression of RESIST-M1 genes (i.e. SERPINE1 and SMARCD3) and low expression RESIST-M2 genes (i.e. SC5D, FDPS, MVD, HMGCS1, HMGCR, CYP51A1, ACAT2 ) while “-RESIST-M” here denotes vice-versa. B, Relapse-free survival (RFS) of patients from publicly available datasets GSE14333, 37892, 39582 stratified using RESIST-M signature. C, RFS of patients from publicly available datasets GSEA14333, 37892, 39582 stratified using each gene in the RESIST-M signature. Red and blue curves denote high and low expression of each gene respectively. D-F, RFS for CRC patients in PETACC-3 dataset using RESIST-M2 gene signature for CMS4 alone (D), CMS1-3 (E), all CMS (F) subtypes. Red and blue curves denote low and high expression of each gene respectively. Statistical significance was determined using log-rank p test.
