# Supplementary material for: Modeling oxaliplatin resistance in colorectal cancer reveals a SERPINE1-based gene signature (RESIST-M) and therapeutic strategies for pro-metastatic CMS4 subtype
Source: Cell Death Dis. 2025 Jul 16;16(1):529. doi: 10.1038/s41419-025-07855-y (PMC12264272; doi:10.1038/s41419-025-07855-y)
Supplement: Supplementary file 6 — Supplementary Figure S6 [file 41419_2025_7855_MOESM6_ESM.pptx]

## Slide 1
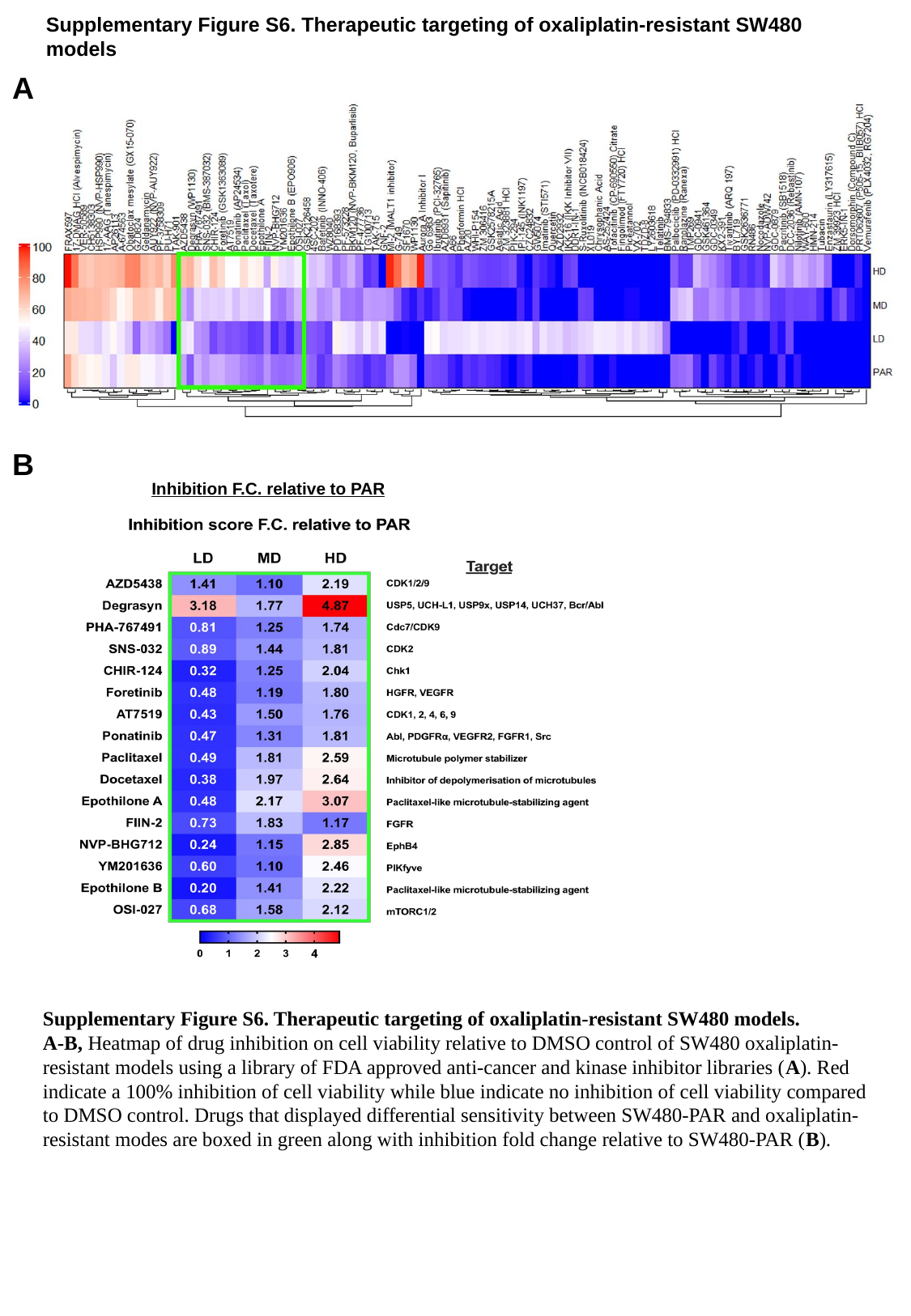

Supplementary Figure S6. Therapeutic targeting of oxaliplatin-resistant SW480 models
A
B
Inhibition F.C. relative to PAR
Supplementary Figure S6. Therapeutic targeting of oxaliplatin-resistant SW480 models.
A-B, Heatmap of drug inhibition on cell viability relative to DMSO control of SW480 oxaliplatin-resistant models using a library of FDA approved anti-cancer and kinase inhibitor libraries (A). Red indicate a 100% inhibition of cell viability while blue indicate no inhibition of cell viability compared to DMSO control. Drugs that displayed differential sensitivity between SW480-PAR and oxaliplatin-resistant modes are boxed in green along with inhibition fold change relative to SW480-PAR (B).
