# Supplementary material for: Modeling oxaliplatin resistance in colorectal cancer reveals a SERPINE1-based gene signature (RESIST-M) and therapeutic strategies for pro-metastatic CMS4 subtype
Source: Cell Death Dis. 2025 Jul 16;16(1):529. doi: 10.1038/s41419-025-07855-y (PMC12264272; doi:10.1038/s41419-025-07855-y)
Supplement: Supplementary file 7 — Original western blots [file 41419_2025_7855_MOESM7_ESM.pptx]

## Slide 1
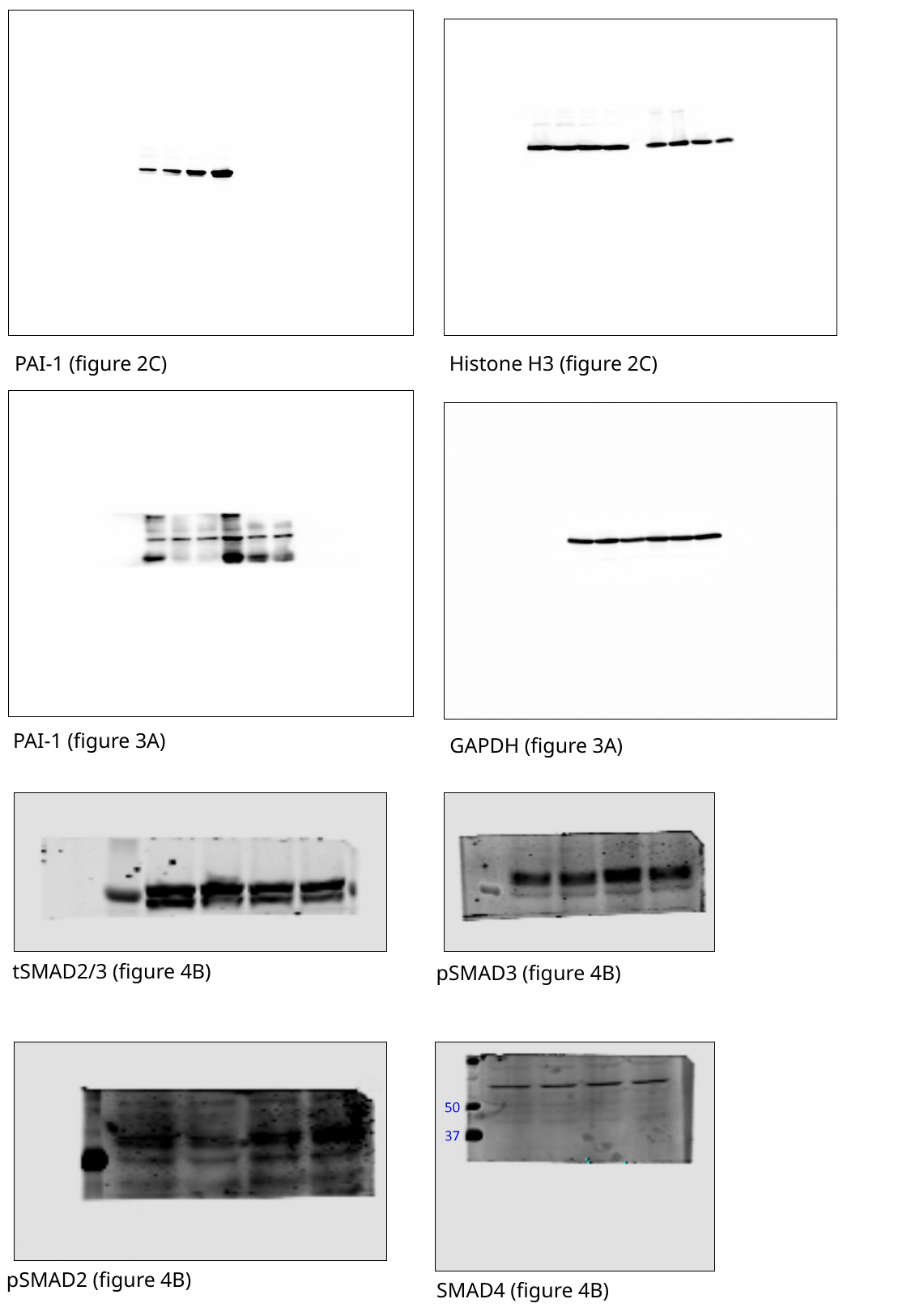

PAI-1 (figure 2C)
Histone H3 (figure 2C)
PAI-1 (figure 3A)
GAPDH (figure 3A)
tSMAD2/3 (figure 4B)
pSMAD3 (figure 4B)
pSMAD2 (figure 4B)
SMAD4 (figure 4B)

## Slide 2
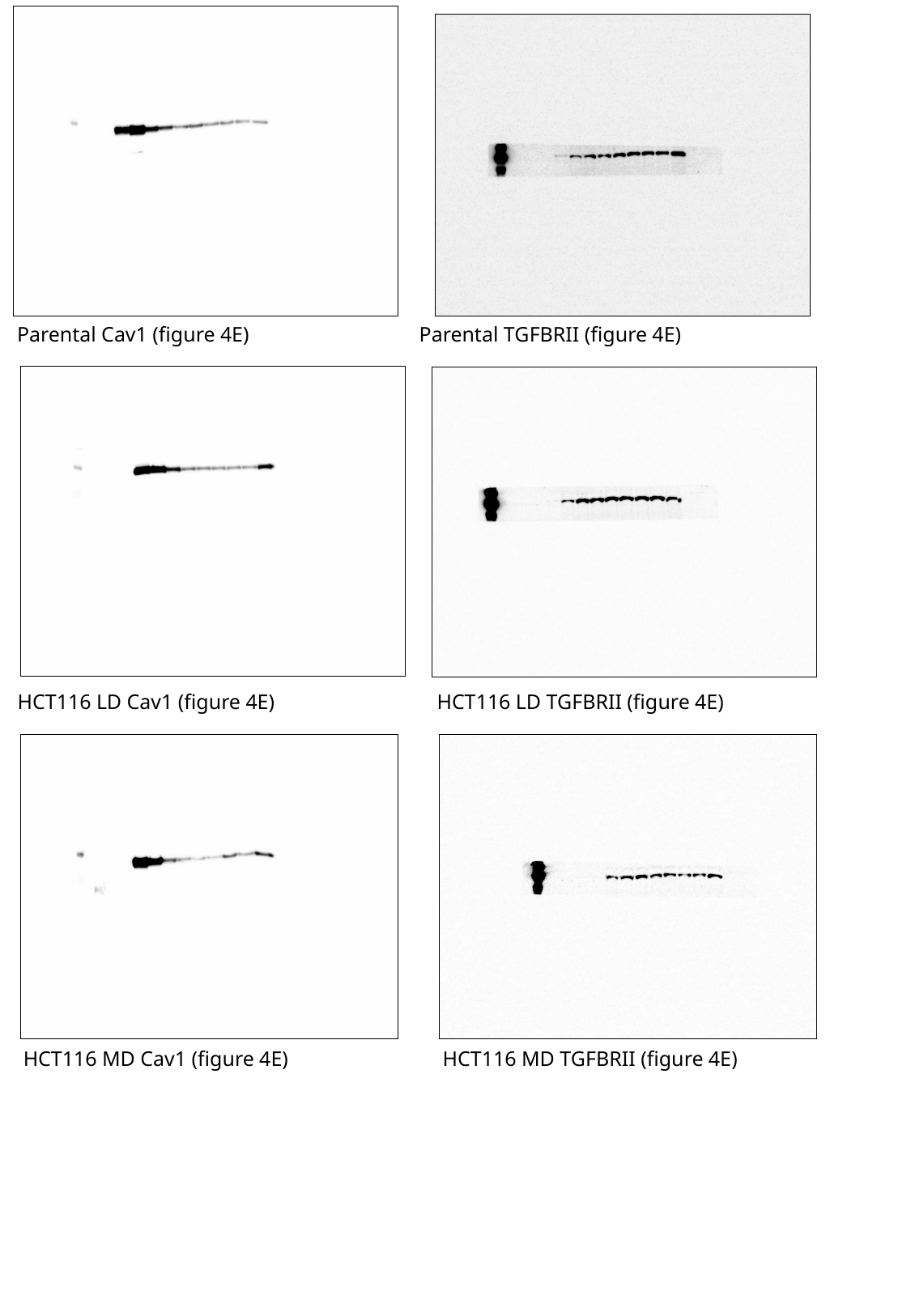

Parental Cav1 (figure 4E)
Parental TGFBRII (figure 4E)
HCT116 LD Cav1 (figure 4E)
HCT116 LD TGFBRII (figure 4E)
HCT116 MD Cav1 (figure 4E)
HCT116 MD TGFBRII (figure 4E)

## Slide 3
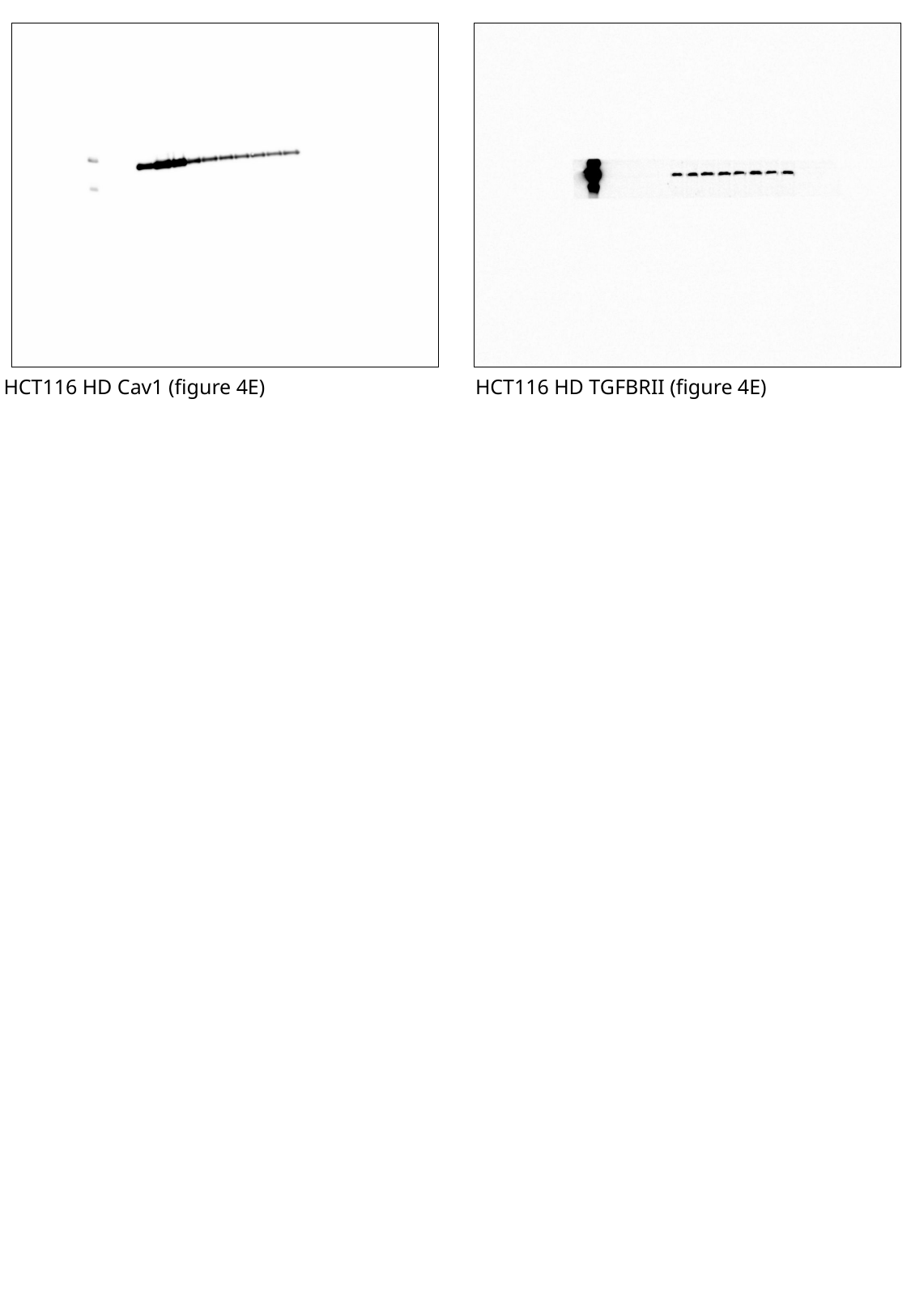

HCT116 HD TGFBRII (figure 4E)
HCT116 HD Cav1 (figure 4E)
